# Measurement of the differential $\hbox {t}\overline{\hbox {t}}$ production cross section as a function of the jet mass and extraction of the top quark mass in hadronic decays of boosted top quarks

**DOI:** 10.1140/epjc/s10052-023-11587-8

**Published:** 2023-07-03

**Authors:** A. Tumasyan, W. Adam, J. W. Andrejkovic, T. Bergauer, S. Chatterjee, K. Damanakis, M. Dragicevic, A. Escalante Del Valle, P. S. Hussain, M. Jeitler, N. Krammer, L. Lechner, D. Liko, I. Mikulec, P. Paulitsch, F. M. Pitters, J. Schieck, R. Schöfbeck, D. Schwarz, S. Templ, W. Waltenberger, C.-E. Wulz, M. R. Darwish, T. Janssen, T. Kello, H. Rejeb Sfar, P. Van Mechelen, E. S. Bols, J. D’Hondt, A. De Moor, M. Delcourt, H. El Faham, S. Lowette, S. Moortgat, A. Morton, D. Müller, A. R. Sahasransu, S. Tavernier, W. Van Doninck, D. Vannerom, B. Clerbaux, G. De Lentdecker, L. Favart, D. Hohov, J. Jaramillo, K. Lee, M. Mahdavikhorrami, I. Makarenko, A. Malara, S. Paredes, L. Pétré, N. Postiau, L. Thomas, M. Vanden Bemden, C. Vander Velde, P. Vanlaer, D. Dobur, J. Knolle, L. Lambrecht, G. Mestdach, M. Niedziela, C. Rendón, C. Roskas, A. Samalan, K. Skovpen, M. Tytgat, N. Van Den Bossche, B. Vermassen, L. Wezenbeek, A. Benecke, G. Bruno, F. Bury, C. Caputo, P. David, C. Delaere, I. S. Donertas, A. Giammanco, K. Jaffel, Sa. Jain, V. Lemaitre, K. Mondal, A. Taliercio, T. T. Tran, P. Vischia, S. Wertz, G. A. Alves, E. Coelho, C. Hensel, A. Moraes, P. Rebello Teles, W. L. Aldá Júnior, M. Alves Gallo Pereira, M. Barroso Ferreira Filho, H. Brandao Malbouisson, W. Carvalho, J. Chinellato, E. M. Da Costa, G. G. Da Silveira, D. De Jesus Damiao, V. Dos Santos Sousa, S. Fonseca De Souza, J. Martins, C. Mora Herrera, K. Mota Amarilo, L. Mundim, H. Nogima, A. Santoro, S. M. Silva Do Amaral, A. Sznajder, M. Thiel, F. Torres Da Silva De Araujo, A. Vilela Pereira, C. A. Bernardes, L. Calligaris, T. R. Fernandez Perez Tomei, E. M. Gregores, P. G. Mercadante, S. F. Novaes, Sandra S. Padula, A. Aleksandrov, G. Antchev, R. Hadjiiska, P. Iaydjiev, M. Misheva, M. Rodozov, M. Shopova, G. Sultanov, A. Dimitrov, T. Ivanov, L. Litov, B. Pavlov, P. Petkov, A. Petrov, E. Shumka, S. Thakur, T. Cheng, T. Javaid, M. Mittal, L. Yuan, M. Ahmad, G. Bauer, Z. Hu, S. Lezki, K. Yi, G. M. Chen, H. S. Chen, M. Chen, F. Iemmi, C. H. Jiang, A. Kapoor, H. Kou, H. Liao, Z.-A. Liu, V. Milosevic, F. Monti, R. Sharma, J. Tao, J. Thomas-Wilsker, J. Wang, H. Zhang, J. Zhao, A. Agapitos, Y. An, Y. Ban, C. Chen, A. Levin, C. Li, Q. Li, X. Lyu, Y. Mao, S. J. Qian, X. Sun, D. Wang, J. Xiao, H. Yang, M. Lu, Z. You, X. Gao, D. Leggat, H. Okawa, Y. Zhang, Z. Lin, C. Lu, M. Xiao, C. Avila, D. A. Barbosa Trujillo, A. Cabrera, C. Florez, J. Fraga, J. Mejia Guisao, F. Ramirez, M. Rodriguez, J. D. Ruiz Alvarez, D. Giljanovic, N. Godinovic, D. Lelas, I. Puljak, Z. Antunovic, M. Kovac, T. Sculac, V. Brigljevic, B. K. Chitroda, D. Ferencek, D. Majumder, S. Mishra, M. Roguljic, A. Starodumov, T. Susa, A. Attikis, K. Christoforou, M. Kolosova, S. Konstantinou, J. Mousa, C. Nicolaou, F. Ptochos, P. A. Razis, H. Rykaczewski, H. Saka, A. Stepennov, M. Finger, M. Finger, A. Kveton, E. Ayala, E. Carrera Jarrin, H. Abdalla, Y. Assran, M. A. Mahmoud, Y. Mohammed, S. Bhowmik, R. K. Dewanjee, K. Ehataht, M. Kadastik, T. Lange, S. Nandan, C. Nielsen, J. Pata, M. Raidal, L. Tani, C. Veelken, P. Eerola, H. Kirschenmann, K. Osterberg, M. Voutilainen, S. Bharthuar, E. Brücken, F. Garcia, J. Havukainen, M. S. Kim, R. Kinnunen, T. Lampén, K. Lassila-Perini, S. Lehti, T. Lindén, M. Lotti, L. Martikainen, M. Myllymäki, J. Ott, M. m. Rantanen, H. Siikonen, E. Tuominen, J. Tuominiemi, P. Luukka, H. Petrow, T. Tuuva, C. Amendola, M. Besancon, F. Couderc, M. Dejardin, D. Denegri, J. L. Faure, F. Ferri, S. Ganjour, P. Gras, G. Hamel de Monchenault, P. Jarry, V. Lohezic, J. Malcles, J. Rander, A. Rosowsky, M. Ö. Sahin, A. Savoy-Navarro, P. Simkina, M. Titov, C. Baldenegro Barrera, F. Beaudette, A. Buchot Perraguin, P. Busson, A. Cappati, C. Charlot, F. Damas, O. Davignon, B. Diab, G. Falmagne, B. A. Fontana Santos Alves, S. Ghosh, R. Granier de Cassagnac, A. Hakimi, B. Harikrishnan, G. Liu, J. Motta, M. Nguyen, C. Ochando, L. Portales, R. Salerno, U. Sarkar, J. B. Sauvan, Y. Sirois, A. Tarabini, E. Vernazza, A. Zabi, A. Zghiche, J.-L. Agram, J. Andrea, D. Apparu, D. Bloch, G. Bourgatte, J.-M. Brom, E. C. Chabert, C. Collard, D. Darej, U. Goerlach, C. Grimault, A.-C. Le Bihan, P. Van Hove, S. Beauceron, B. Blancon, G. Boudoul, A. Carle, N. Chanon, J. Choi, D. Contardo, P. Depasse, C. Dozen, H. El Mamouni, J. Fay, S. Gascon, M. Gouzevitch, G. Grenier, B. Ille, I. B. Laktineh, M. Lethuillier, L. Mirabito, S. Perries, L. Torterotot, M. Vander Donckt, P. Verdier, S. Viret, I. Lomidze, T. Toriashvili, Z. Tsamalaidze, V. Botta, L. Feld, K. Klein, M. Lipinski, D. Meuser, A. Pauls, N. Röwert, M. Teroerde, S. Diekmann, A. Dodonova, N. Eich, D. Eliseev, M. Erdmann, P. Fackeldey, D. Fasanella, B. Fischer, T. Hebbeker, K. Hoepfner, F. Ivone, M. y. Lee, L. Mastrolorenzo, M. Merschmeyer, A. Meyer, S. Mondal, S. Mukherjee, D. Noll, A. Novak, F. Nowotny, A. Pozdnyakov, Y. Rath, W. Redjeb, H. Reithler, A. Schmidt, S. C. Schuler, A. Sharma, L. Vigilante, S. Wiedenbeck, S. Zaleski, C. Dziwok, G. Flügge, W. Haj Ahmad, O. Hlushchenko, T. Kress, A. Nowack, O. Pooth, A. Stahl, T. Ziemons, A. Zotz, H. Aarup Petersen, M. Aldaya Martin, P. Asmuss, S. Baxter, M. Bayatmakou, O. Behnke, A. Bermúdez Martínez, S. Bhattacharya, A. A. Bin Anuar, F. Blekman, K. Borras, D. Brunner, A. Campbell, A. Cardini, C. Cheng, F. Colombina, S. Consuegra Rodríguez, G. Correia Silva, M. De Silva, L. Didukh, G. Eckerlin, D. Eckstein, L. I. Estevez Banos, O. Filatov, E. Gallo, A. Geiser, A. Giraldi, G. Greau, A. Grohsjean, V. Guglielmi, M. Guthoff, A. Jafari, N. Z. Jomhari, B. Kaech, A. Kasem, M. Kasemann, H. Kaveh, C. Kleinwort, R. Kogler, M. Komm, D. Krücker, W. Lange, D. Leyva Pernia, K. Lipka, W. Lohmann, R. Mankel, I. -A. Melzer-Pellmann, M. Mendizabal Morentin, J. Metwally, A. B. Meyer, G. Milella, M. Mormile, A. Mussgiller, A. Nürnberg, Y. Otarid, D. Pérez Adán, A. Raspereza, B. Ribeiro Lopes, J. Rübenach, A. Saggio, A. Saibel, M. Savitskyi, M. Scham, V. Scheurer, S. Schnake, P. Schütze, C. Schwanenberger, M. Shchedrolosiev, R. E. Sosa Ricardo, D. Stafford, N. Tonon, M. Van De Klundert, F. Vazzoler, A. Ventura Barroso, R. Walsh, D. Walter, Q. Wang, Y. Wen, K. Wichmann, L. Wiens, C. Wissing, S. Wuchterl, Y. Yang, A. Zimermmane Castro Santos, A. Albrecht, S. Albrecht, M. Antonello, S. Bein, L. Benato, M. Bonanomi, P. Connor, K. De Leo, M. Eich, K. El Morabit, F. Feindt, A. Fröhlich, C. Garbers, E. Garutti, M. Hajheidari, J. Haller, A. Hinzmann, H. R. Jabusch, G. Kasieczka, P. Keicher, R. Klanner, W. Korcari, T. Kramer, V. Kutzner, F. Labe, J. Lange, A. Lobanov, C. Matthies, A. Mehta, L. Moureaux, M. Mrowietz, A. Nigamova, Y. Nissan, A. Paasch, K. J. Pena Rodriguez, T. Quadfasel, M. Rieger, O. Rieger, D. Savoiu, P. Schleper, M. Schröder, J. Schwandt, M. Sommerhalder, H. Stadie, G. Steinbrück, A. Tews, M. Wolf, S. Brommer, M. Burkart, E. Butz, R. Caspart, T. Chwalek, A. Dierlamm, A. Droll, N. Faltermann, M. Giffels, J. O. Gosewisch, A. Gottmann, F. Hartmann, M. Horzela, U. Husemann, M. Klute, R. Koppenhöfer, S. Maier, S. Mitra, Th. Müller, M. Neukum, M. Oh, G. Quast, K. Rabbertz, J. Rauser, M. Schnepf, D. Seith, I. Shvetsov, H. J. Simonis, N. Trevisani, R. Ulrich, J. van der Linden, R. F. Von Cube, M. Wassmer, S. Wieland, R. Wolf, S. Wozniewski, S. Wunsch, X. Zuo, G. Anagnostou, P. Assiouras, G. Daskalakis, A. Kyriakis, A. Stakia, M. Diamantopoulou, D. Karasavvas, P. Kontaxakis, A. Manousakis-Katsikakis, A. Panagiotou, I. Papavergou, N. Saoulidou, K. Theofilatos, E. Tziaferi, K. Vellidis, I. Zisopoulos, G. Bakas, T. Chatzistavrou, K. Kousouris, I. Papakrivopoulos, G. Tsipolitis, A. Zacharopoulou, K. Adamidis, I. Bestintzanos, I. Evangelou, C. Foudas, P. Gianneios, C. Kamtsikis, P. Katsoulis, P. Kokkas, P. G. Kosmoglou Kioseoglou, N. Manthos, I. Papadopoulos, J. Strologas, M. Csanád, K. Farkas, M. M. A. Gadallah, S. Lökös, P. Major, K. Mandal, G. Pásztor, A. J. Rádl, O. Surányi, G. I. Veres, M. Bartók, G. Bencze, C. Hajdu, D. Horvath, F. Sikler, V. Veszpremi, N. Beni, S. Czellar, J. Karancsi, J. Molnar, Z. Szillasi, D. Teyssier, P. Raics, B. Ujvari, T. Csorgo, F. Nemes, T. Novak, J. Babbar, S. Bansal, S. B. Beri, V. Bhatnagar, G. Chaudhary, S. Chauhan, N. Dhingra, R. Gupta, A. Kaur, A. Kaur, H. Kaur, M. Kaur, S. Kumar, P. Kumari, M. Meena, K. Sandeep, T. Sheokand, J. B. Singh, A. Singla, A. K. Virdi, A. Ahmed, A. Bhardwaj, B. C. Choudhary, A. Kumar, M. Naimuddin, K. Ranjan, S. Saumya, S. Baradia, S. Barman, S. Bhattacharya, D. Bhowmik, S. Dutta, S. Dutta, B. Gomber, M. Maity, P. Palit, G. Saha, B. Sahu, S. Sarkar, P. K. Behera, S. C. Behera, P. Kalbhor, J. R. Komaragiri, D. Kumar, A. Muhammad, L. Panwar, R. Pradhan, P. R. Pujahari, A. Sharma, A. K. Sikdar, P. C. Tiwari, S. Verma, K. Naskar, T. Aziz, I. Das, S. Dugad, M. Kumar, G. B. Mohanty, P. Suryadevara, S. Banerjee, R. Chudasama, M. Guchait, S. Karmakar, S. Kumar, G. Majumder, K. Mazumdar, S. Mukherjee, A. Thachayath, S. Bahinipati, C. Kar, P. Mal, T. Mishra, V. K. Muraleedharan Nair Bindhu, A. Nayak, P. Saha, S. K. Swain, D. Vats, A. Alpana, S. Dube, B. Kansal, A. Laha, S. Pandey, A. Rastogi, S. Sharma, H. Bakhshiansohi, E. Khazaie, M. Zeinali, S. Chenarani, S. M. Etesami, M. Khakzad, M. Mohammadi Najafabadi, M. Grunewald, M. Abbrescia, R. Aly, C. Aruta, A. Colaleo, D. Creanza, N. De Filippis, M. De Palma, A. Di Florio, W. Elmetenawee, F. Errico, L. Fiore, G. Iaselli, M. Ince, G. Maggi, M. Maggi, I. Margjeka, V. Mastrapasqua, S. My, S. Nuzzo, A. Pellecchia, A. Pompili, G. Pugliese, R. Radogna, D. Ramos, A. Ranieri, G. Selvaggi, L. Silvestris, F. M. Simone, Ü. Sözbilir, A. Stamerra, R. Venditti, P. Verwilligen, G. Abbiendi, C. Battilana, D. Bonacorsi, L. Borgonovi, L. Brigliadori, R. Campanini, P. Capiluppi, A. Castro, F. R. Cavallo, M. Cuffiani, G. M. Dallavalle, T. Diotalevi, F. Fabbri, A. Fanfani, P. Giacomelli, L. Giommi, C. Grandi, L. Guiducci, S. Lo Meo, L. Lunerti, S. Marcellini, G. Masetti, F. L. Navarria, A. Perrotta, F. Primavera, A. M. Rossi, T. Rovelli, G. P. Siroli, S. Costa, A. Di Mattia, R. Potenza, A. Tricomi, C. Tuve, G. Barbagli, G. Bardelli, B. Camaiani, A. Cassese, R. Ceccarelli, V. Ciulli, C. Civinini, R. D’Alessandro, E. Focardi, G. Latino, P. Lenzi, M. Lizzo, M. Meschini, S. Paoletti, R. Seidita, G. Sguazzoni, L. Viliani, L. Benussi, S. Bianco, S. Meola, D. Piccolo, M. Bozzo, P. Chatagnon, F. Ferro, R. Mulargia, E. Robutti, S. Tosi, A. Benaglia, G. Boldrini, F. Brivio, F. Cetorelli, F. De Guio, M. E. Dinardo, P. Dini, S. Gennai, A. Ghezzi, P. Govoni, L. Guzzi, M. T. Lucchini, M. Malberti, S. Malvezzi, A. Massironi, D. Menasce, L. Moroni, M. Paganoni, D. Pedrini, B. S. Pinolini, S. Ragazzi, N. Redaelli, T. Tabarelli de Fatis, D. Zuolo, S. Buontempo, F. Carnevali, N. Cavallo, A. De Iorio, F. Fabozzi, A. O. M. Iorio, L. Lista, P. Paolucci, B. Rossi, C. Sciacca, P. Azzi, N. Bacchetta, M. Bellato, D. Bisello, P. Bortignon, A. Bragagnolo, R. Carlin, P. Checchia, T. Dorigo, G. Grosso, L. Layer, E. Lusiani, M. Margoni, A. T. Meneguzzo, J. Pazzini, P. Ronchese, R. Rossin, F. Simonetto, G. Strong, M. Tosi, S. Ventura, H. Yarar, M. Zanetti, P. Zotto, A. Zucchetta, G. Zumerle, S. Abu Zeid, C. Aimè, A. Braghieri, S. Calzaferri, D. Fiorina, P. Montagna, V. Re, C. Riccardi, P. Salvini, I. Vai, P. Vitulo, P. Asenov, G. M. Bilei, D. Ciangottini, L. Fanò, M. Magherini, G. Mantovani, V. Mariani, M. Menichelli, F. Moscatelli, A. Piccinelli, M. Presilla, A. Rossi, A. Santocchia, D. Spiga, T. Tedeschi, P. Azzurri, G. Bagliesi, V. Bertacchi, R. Bhattacharya, L. Bianchini, T. Boccali, E. Bossini, D. Bruschini, R. Castaldi, M. A. Ciocci, V. D’Amante, R. Dell’Orso, M. R. Di Domenico, S. Donato, A. Giassi, F. Ligabue, G. Mandorli, D. Matos Figueiredo, A. Messineo, M. Musich, F. Palla, S. Parolia, G. Ramirez-Sanchez, A. Rizzi, G. Rolandi, S. Roy Chowdhury, T. Sarkar, A. Scribano, N. Shafiei, P. Spagnolo, R. Tenchini, G. Tonelli, N. Turini, A. Venturi, P. G. Verdini, P. Barria, M. Campana, F. Cavallari, D. Del Re, E. Di Marco, M. Diemoz, E. Longo, P. Meridiani, G. Organtini, F. Pandolfi, R. Paramatti, C. Quaranta, S. Rahatlou, C. Rovelli, F. Santanastasio, L. Soffi, R. Tramontano, N. Amapane, R. Arcidiacono, S. Argiro, M. Arneodo, N. Bartosik, R. Bellan, A. Bellora, C. Biino, N. Cartiglia, M. Costa, R. Covarelli, N. Demaria, M. Grippo, B. Kiani, F. Legger, C. Mariotti, S. Maselli, A. Mecca, E. Migliore, E. Monteil, M. Monteno, M. M. Obertino, G. Ortona, L. Pacher, N. Pastrone, M. Pelliccioni, M. Ruspa, K. Shchelina, F. Siviero, V. Sola, A. Solano, D. Soldi, A. Staiano, M. Tornago, D. Trocino, G. Umoret, A. Vagnerini, S. Belforte, V. Candelise, M. Casarsa, F. Cossutti, A. Da Rold, G. Della Ricca, G. Sorrentino, S. Dogra, C. Huh, B. Kim, D. H. Kim, G. N. Kim, J. Kim, J. Lee, S. W. Lee, C. S. Moon, Y. D. Oh, S. I. Pak, M. S. Ryu, S. Sekmen, Y. C. Yang, H. Kim, D. H. Moon, E. Asilar, T. J. Kim, J. Park, S. Choi, S. Han, B. Hong, K. Lee, K. S. Lee, J. Lim, J. Park, S. K. Park, J. Yoo, J. Goh, H. S. Kim, Y. Kim, S. Lee, J. Almond, J. H. Bhyun, J. Choi, S. Jeon, J. Kim, J. S. Kim, S. Ko, H. Kwon, H. Lee, S. Lee, B. H. Oh, S. B. Oh, H. Seo, U. K. Yang, I. Yoon, W. Jang, D. Y. Kang, Y. Kang, D. Kim, S. Kim, B. Ko, J. S. H. Lee, Y. Lee, J. A. Merlin, I. C. Park, Y. Roh, D. Song, Watson I. J., S. Yang, S. Ha, H. D. Yoo, M. Choi, M. R. Kim, H. Lee, Y. Lee, Y. Lee, I. Yu, T. Beyrouthy, Y. Maghrbi, K. Dreimanis, G. Pikurs, M. Seidel, V. Veckalns, M. Ambrozas, A. Carvalho Antunes De Oliveira, A. Juodagalvis, A. Rinkevicius, G. Tamulaitis, N. Bin Norjoharuddeen, S. Y. Hoh, I. Yusuff, Z. Zolkapli, J. F. Benitez, A. Castaneda Hernandez, H. A. Encinas Acosta, L. G. Gallegos Maríñez, M. León Coello, J. A. Murillo Quijada, A. Sehrawat, L. Valencia Palomo, G. Ayala, H. Castilla-Valdez, I. Heredia-De La Cruz, R. Lopez-Fernandez, C. A. Mondragon Herrera, D. A. Perez Navarro, A. Sánchez Hernández, C. Oropeza Barrera, F. Vazquez Valencia, I. Pedraza, H. A. Salazar Ibarguen, C. Uribe Estrada, I. Bubanja, J. Mijuskovic, N. Raicevic, A. Ahmad, M. I. Asghar, A. Awais, M. I. M. Awan, M. Gul, H. R. Hoorani, W. A. Khan, M. Shoaib, M. Waqas, V. Avati, L. Grzanka, M. Malawski, H. Bialkowska, M. Bluj, B. Boimska, M. Górski, M. Kazana, M. Szleper, P. Zalewski, K. Bunkowski, K. Doroba, A. Kalinowski, M. Konecki, J. Krolikowski, M. Araujo, P. Bargassa, D. Bastos, A. Boletti, P. Faccioli, M. Gallinaro, J. Hollar, N. Leonardo, T. Niknejad, M. Pisano, J. Seixas, J. Varela, P. Adzic, M. Dordevic, P. Milenovic, J. Milosevic, M. Aguilar-Benitez, J. Alcaraz Maestre, A. Álvarez Fernández, M. Barrio Luna, Cristina F. Bedoya, C. A. Carrillo Montoya, M. Cepeda, M. Cerrada, N. Colino, B. De La Cruz, A. Delgado Peris, D. Fernández Del Val, J. P. Fernández Ramos, J. Flix, M. C. Fouz, O. Gonzalez Lopez, S. Goy Lopez, J. M. Hernandez, M. I. Josa, J. León Holgado, D. Moran, C. Perez Dengra, A. Pérez-Calero Yzquierdo, J. Puerta Pelayo, I. Redondo, D. D. Redondo Ferrero, L. Romero, S. Sánchez Navas, J. Sastre, L. Urda Gómez, J. Vazquez Escobar, C. Willmott, J. F. de Trocóniz, B. Alvarez Gonzalez, J. Cuevas, J. Fernandez Menendez, S. Folgueras, I. Gonzalez Caballero, J. R. González Fernández, E. Palencia Cortezon, C. Ramón Álvarez, V. Rodríguez Bouza, A. Soto Rodríguez, A. Trapote, C. Vico Villalba, J. A. Brochero Cifuentes, I. J. Cabrillo, A. Calderon, J. Duarte Campderros, M. Fernandez, C. Fernandez Madrazo, A. García Alonso, G. Gomez, C. Lasaosa García, C. Martinez Rivero, P. Martinez Ruiz del Arbol, F. Matorras, P. Matorras Cuevas, J. Piedra Gomez, C. Prieels, A. Ruiz-Jimeno, L. Scodellaro, I. Vila, J. M. Vizan Garcia, M. K. Jayananda, B. Kailasapathy, D. U. J. Sonnadara, D. D. C. Wickramarathna, W. G. D. Dharmaratna, K. Liyanage, N. Perera, N. Wickramage, D. Abbaneo, J. Alimena, E. Auffray, G. Auzinger, J. Baechler, P. Baillon, D. Barney, J. Bendavid, M. Bianco, B. Bilin, A. Bocci, E. Brondolin, C. Caillol, T. Camporesi, G. Cerminara, N. Chernyavskaya, S. S. Chhibra, S. Choudhury, M. Cipriani, L. Cristella, D. d’Enterria, A. Dabrowski, A. David, A. De Roeck, M. M. Defranchis, M. Deile, M. Dobson, M. Dünser, N. Dupont, F. Fallavollita, A. Florent, L. Forthomme, G. Franzoni, W. Funk, S. Ghosh, S. Giani, D. Gigi, K. Gill, F. Glege, L. Gouskos, E. Govorkova, M. Haranko, J. Hegeman, V. Innocente, T. James, P. Janot, J. Kaspar, J. Kieseler, N. Kratochwil, S. Laurila, P. Lecoq, E. Leutgeb, A. Lintuluoto, C. Lourenço, B. Maier, L. Malgeri, M. Mannelli, A. C. Marini, F. Meijers, S. Mersi, E. Meschi, F. Moortgat, M. Mulders, S. Orfanelli, L. Orsini, F. Pantaleo, E. Perez, M. Peruzzi, A. Petrilli, G. Petrucciani, A. Pfeiffer, M. Pierini, D. Piparo, M. Pitt, H. Qu, T. Quast, D. Rabady, A. Racz, G. Reales Gutiérrez, M. Rovere, H. Sakulin, J. Salfeld-Nebgen, S. Scarfi, M. Selvaggi, A. Sharma, P. Silva, P. Sphicas, A. G. Stahl Leiton, S. Summers, K. Tatar, V. R. Tavolaro, D. Treille, P. Tropea, A. Tsirou, J. Wanczyk, K. A. Wozniak, W. D. Zeuner, L. Caminada, A. Ebrahimi, W. Erdmann, R. Horisberger, Q. Ingram, H. C. Kaestli, D. Kotlinski, C. Lange, M. Missiroli, L. Noehte, T. Rohe, T. K. Aarrestad, K. Androsov, M. Backhaus, P. Berger, A. Calandri, K. Datta, A. De Cosa, G. Dissertori, M. Dittmar, M. Donegà, F. Eble, M. Galli, K. Gedia, F. Glessgen, T. A. Gómez Espinosa, C. Grab, D. Hits, W. Lustermann, A.-M. Lyon, R. A. Manzoni, L. Marchese, C. Martin Perez, A. Mascellani, F. Nessi-Tedaldi, J. Niedziela, F. Pauss, V. Perovic, S. Pigazzini, M. G. Ratti, M. Reichmann, C. Reissel, T. Reitenspiess, B. Ristic, F. Riti, D. Ruini, D. A. Sanz Becerra, J. Steggemann, D. Valsecchi, R. Wallny, C. Amsler, P. Bärtschi, C. Botta, D. Brzhechko, M. F. Canelli, K. Cormier, A. De Wit, R. Del Burgo, J. K. Heikkilä, M. Huwiler, W. Jin, A. Jofrehei, B. Kilminster, S. Leontsinis, S. P. Liechti, A. Macchiolo, P. Meiring, V. M. Mikuni, U. Molinatti, I. Neutelings, A. Reimers, P. Robmann, S. Sanchez Cruz, K. Schweiger, M. Senger, Y. Takahashi, C. Adloff, C. M. Kuo, W. Lin, P. K. Rout, S. S. Yu, L. Ceard, Y. Chao, K. F. Chen, P. s. Chen, H. Cheng, W.-S. Hou, R. Khurana, G. Kole, Y. y. Li, R.-S. Lu, E. Paganis, A. Psallidas, A. Steen, H. y. Wu, E. Yazgan, P. r. Yu, C. Asawatangtrakuldee, N. Srimanobhas, D. Agyel, F. Boran, Z. S. Demiroglu, F. Dolek, I. Dumanoglu, E. Eskut, Y. Guler, E. Gurpinar Guler, C. Isik, O. Kara, A. Kayis Topaksu, U. Kiminsu, G. Onengut, K. Ozdemir, A. Polatoz, A. E. Simsek, B. Tali, U. G. Tok, S. Turkcapar, E. Uslan, I. S. Zorbakir, G. Karapinar, K. Ocalan, M. Yalvac, B. Akgun, I. O. Atakisi, E. Gülmez, M. Kaya, O. Kaya, S. Tekten, A. Cakir, K. Cankocak, Y. Komurcu, S. Sen, O. Aydilek, S. Cerci, B. Hacisahinoglu, I. Hos, B. Isildak, B. Kaynak, S. Ozkorucuklu, C. Simsek, D. Sunar Cerci, B. Grynyov, L. Levchuk, D. Anthony, E. Bhal, J. J. Brooke, A. Bundock, E. Clement, D. Cussans, H. Flacher, M. Glowacki, J. Goldstein, G. P. Heath, H. F. Heath, L. Kreczko, B. Krikler, S. Paramesvaran, S. Seif El Nasr-Storey, V. J. Smith, N. Stylianou, K. Walkingshaw Pass, R. White, A. H. Ball, K. W. Bell, A. Belyaev, C. Brew, R. M. Brown, D. J. A. Cockerill, C. Cooke, K. V. Ellis, K. Harder, S. Harper, M.-L. Holmberg, J. Linacre, K. Manolopoulos, D. M. Newbold, E. Olaiya, D. Petyt, T. Reis, G. Salvi, T. Schuh, C. H. Shepherd-Themistocleous, I. R. Tomalin, T. Williams, R. Bainbridge, P. Bloch, S. Bonomally, J. Borg, S. Breeze, C. E. Brown, O. Buchmuller, V. Cacchio, V. Cepaitis, G. S. Chahal, D. Colling, J. S. Dancu, P. Dauncey, G. Davies, J. Davies, M. Della Negra, S. Fayer, G. Fedi, G. Hall, M. H. Hassanshahi, A. Howard, G. Iles, J. Langford, L. Lyons, A.-M. Magnan, S. Malik, A. Martelli, M. Mieskolainen, D. G. Monk, J. Nash, M. Pesaresi, B. C. Radburn-Smith, D. M. Raymond, A. Richards, A. Rose, E. Scott, C.-M. Seez, A. Shtipliyski, R. Shukla, A. Tapper, K. Uchida, G. P. Uttley, L. H. Vage, T. Virdee, M. Vojinovic, N. Wardle, S. N. Webb, D. Winterbottom, K. Coldham, J. E. Cole, A. Khan, P. Kyberd, I. D. Reid, S. Abdullin, A. Brinkerhoff, B. Caraway, J. Dittmann, K. Hatakeyama, A. R. Kanuganti, B. McMaster, M. Saunders, S. Sawant, C. Sutantawibul, J. Wilson, R. Bartek, A. Dominguez, R. Uniyal, A. M. Vargas Hernandez, S. I. Cooper, D. Di Croce, S. V. Gleyzer, C. Henderson, C. U. Perez, P. Rumerio, C. West, A. Akpinar, A. Albert, D. Arcaro, C. Cosby, Z. Demiragli, C. Erice, E. Fontanesi, D. Gastler, S. May, J. Rohlf, K. Salyer, D. Sperka, D. Spitzbart, I. Suarez, A. Tsatsos, S. Yuan, G. Benelli, B. Burkle, X. Coubez, D. Cutts, M. Hadley, U. Heintz, J. M. Hogan, T. Kwon, G. Landsberg, K. T. Lau, D. Li, J. Luo, M. Narain, N. Pervan, S. Sagir, F. Simpson, E. Usai, W. Y. Wong, X. Yan, D. Yu, W. Zhang, J. Bonilla, C. Brainerd, R. Breedon, M. Calderon De La Barca Sanchez, M. Chertok, J. Conway, P. T. Cox, R. Erbacher, G. Haza, F. Jensen, O. Kukral, G. Mocellin, M. Mulhearn, D. Pellett, B. Regnery, Y. Yao, F. Zhang, M. Bachtis, R. Cousins, A. Datta, D. Hamilton, J. Hauser, M. Ignatenko, M. A. Iqbal, T. Lam, E. Manca, W. A. Nash, S. Regnard, D. Saltzberg, B. Stone, V. Valuev, R. Clare, J. W. Gary, M. Gordon, G. Hanson, G. Karapostoli, O. R. Long, N. Manganelli, W. Si, S. Wimpenny, J. G. Branson, P. Chang, S. Cittolin, S. Cooperstein, D. Diaz, J. Duarte, R. Gerosa, L. Giannini, J. Guiang, R. Kansal, V. Krutelyov, R. Lee, J. Letts, M. Masciovecchio, F. Mokhtar, M. Pieri, B. V. Sathia Narayanan, V. Sharma, M. Tadel, E. Vourliotis, F. Würthwein, Y. Xiang, A. Yagil, N. Amin, C. Campagnari, M. Citron, G. Collura, A. Dorsett, V. Dutta, J. Incandela, M. Kilpatrick, J. Kim, A. J. Li, P. Masterson, H. Mei, M. Oshiro, M. Quinnan, J. Richman, U. Sarica, R. Schmitz, F. Setti, J. Sheplock, P. Siddireddy, D. Stuart, S. Wang, A. Bornheim, O. Cerri, I. Dutta, A. Latorre, J. M. Lawhorn, N. Lu, J. Mao, H. B. Newman, T. Q. Nguyen, M. Spiropulu, J. R. Vlimant, C. Wang, S. Xie, R. Y. Zhu, J. Alison, S. An, M. B. Andrews, P. Bryant, T. Ferguson, A. Harilal, C. Liu, T. Mudholkar, S. Murthy, M. Paulini, A. Roberts, A. Sanchez, W. Terrill, J. P. Cumalat, W. T. Ford, A. Hassani, G. Karathanasis, E. MacDonald, F. Marini, R. Patel, A. Perloff, C. Savard, N. Schonbeck, K. Stenson, K. A. Ulmer, S. R. Wagner, N. Zipper, J. Alexander, S. Bright-Thonney, X. Chen, D. J. Cranshaw, J. Fan, X. Fan, D. Gadkari, S. Hogan, J. Monroy, J. R. Patterson, D. Quach, J. Reichert, M. Reid, A. Ryd, J. Thom, P. Wittich, R. Zou, M. Albrow, M. Alyari, G. Apollinari, A. Apresyan, L. A. T. Bauerdick, D. Berry, J. Berryhill, P. C. Bhat, K. Burkett, J. N. Butler, A. Canepa, G. B. Cerati, H. W. K. Cheung, F. Chlebana, K. F. Di Petrillo, J. Dickinson, V. D. Elvira, Y. Feng, J. Freeman, A. Gandrakota, Z. Gecse, L. Gray, D. Green, S. Grünendahl, O. Gutsche, R. M. Harris, R. Heller, T. C. Herwig, J. Hirschauer, L. Horyn, B. Jayatilaka, S. Jindariani, M. Johnson, U. Joshi, T. Klijnsma, B. Klima, K. H. M. Kwok, S. Lammel, D. Lincoln, R. Lipton, T. Liu, C. Madrid, K. Maeshima, C. Mantilla, D. Mason, P. McBride, P. Merkel, S. Mrenna, S. Nahn, J. Ngadiuba, D. Noonan, V. Papadimitriou, N. Pastika, K. Pedro, C. Pena, F. Ravera, A. Reinsvold Hall, L. Ristori, E. Sexton-Kennedy, N. Smith, A. Soha, L. Spiegel, J. Strait, L. Taylor, S. Tkaczyk, N. V. Tran, L. Uplegger, E. W. Vaandering, H. A. Weber, I. Zoi, P. Avery, D. Bourilkov, L. Cadamuro, V. Cherepanov, R. D. Field, D. Guerrero, M. Kim, E. Koenig, J. Konigsberg, A. Korytov, K. H. Lo, K. Matchev, N. Menendez, G. Mitselmakher, A. Muthirakalayil Madhu, N. Rawal, D. Rosenzweig, S. Rosenzweig, K. Shi, J. Wang, Z. Wu, T. Adams, A. Askew, R. Habibullah, V. Hagopian, T. Kolberg, G. Martinez, H. Prosper, C. Schiber, O. Viazlo, R. Yohay, J. Zhang, M. M. Baarmand, S. Butalla, T. Elkafrawy, M. Hohlmann, R. Kumar Verma, M. Rahmani, F. Yumiceva, M. R. Adams, H. Becerril Gonzalez, R. Cavanaugh, S. Dittmer, O. Evdokimov, C. E. Gerber, D. J. Hofman, D. S. Lemos, A. H. Merrit, C. Mills, G. Oh, T. Roy, S. Rudrabhatla, M. B. Tonjes, N. Varelas, X. Wang, Z. Ye, J. Yoo, M. Alhusseini, K. Dilsiz, L. Emediato, R. P. Gandrajula, G. Karaman, O. K. Köseyan, J. -P. Merlo, A. Mestvirishvili, J. Nachtman, O. Neogi, H. Ogul, Y. Onel, A. Penzo, C. Snyder, E. Tiras, O. Amram, B. Blumenfeld, L. Corcodilos, J. Davis, A. V. Gritsan, S. Kyriacou, P. Maksimovic, J. Roskes, S. Sekhar, M. Swartz, T. Á. Vámi, A. Abreu, L. F. Alcerro Alcerro, J. Anguiano, P. Baringer, A. Bean, Z. Flowers, T. Isidori, J. King, G. Krintiras, M. Lazarovits, C. Le Mahieu, C. Lindsey, J. Marquez, N. Minafra, M. Murray, M. Nickel, C. Rogan, C. Royon, R. Salvatico, S. Sanders, C. Smith, Q. Wang, J. Williams, G. Wilson, B. Allmond, S. Duric, A. Ivanov, K. Kaadze, D. Kim, Y. Maravin, T. Mitchell, A. Modak, K. Nam, D. Roy, F. Rebassoo, D. Wright, E. Adams, A. Baden, O. Baron, A. Belloni, A. Bethani, S. C. Eno, N. J. Hadley, S. Jabeen, R. G. Kellogg, T. Koeth, Y. Lai, S. Lascio, A. C. Mignerey, S. Nabili, C. Palmer, C. Papageorgakis, L. Wang, K. Wong, D. Abercrombie, W. Busza, I. A. Cali, Y. Chen, M. D’Alfonso, J. Eysermans, C. Freer, G. Gomez-Ceballos, M. Goncharov, P. Harris, M. Hu, D. Kovalskyi, J. Krupa, Y.-J. Lee, K. Long, C. Mironov, C. Paus, D. Rankin, C. Roland, G. Roland, Z. Shi, G. S. F. Stephans, J. Wang, Z. Wang, B. Wyslouch, T. J. Yang, R. M. Chatterjee, B. Crossman, A. Evans, J. Hiltbrand, Sh. Jain, B. M. Joshi, C. Kapsiak, M. Krohn, Y. Kubota, J. Mans, M. Revering, R. Rusack, R. Saradhy, N. Schroeder, N. Strobbe, M. A. Wadud, L. M. Cremaldi, K. Bloom, M. Bryson, D. R. Claes, C. Fangmeier, L. Finco, F. Golf, C. Joo, R. Kamalieddin, I. Kravchenko, I. Reed, J. E. Siado, G. R. Snow, W. Tabb, A. Wightman, F. Yan, A. G. Zecchinelli, G. Agarwal, H. Bandyopadhyay, L. Hay, I. Iashvili, A. Kharchilava, C. McLean, M. Morris, D. Nguyen, J. Pekkanen, S. Rappoccio, A. Williams, G. Alverson, E. Barberis, Y. Haddad, Y. Han, A. Krishna, J. Li, J. Lidrych, G. Madigan, B. Marzocchi, D. M. Morse, V. Nguyen, T. Orimoto, A. Parker, L. Skinnari, A. Tishelman-Charny, T. Wamorkar, B. Wang, A. Wisecarver, D. Wood, S. Bhattacharya, J. Bueghly, Z. Chen, A. Gilbert, K. A. Hahn, Y. Liu, N. Odell, M. H. Schmitt, M. Velasco, R. Band, R. Bucci, M. Cremonesi, A. Das, R. Goldouzian, M. Hildreth, K. Hurtado Anampa, C. Jessop, K. Lannon, J. Lawrence, N. Loukas, L. Lutton, J. Mariano, N. Marinelli, I. Mcalister, T. McCauley, C. Mcgrady, K. Mohrman, C. Moore, Y. Musienko, R. Ruchti, A. Townsend, M. Wayne, H. Yockey, M. Zarucki, L. Zygala, B. Bylsma, M. Carrigan, L. S. Durkin, B. Francis, C. Hill, M. Joyce, A. Lesauvage, M. Nunez Ornelas, K. Wei, B. L. Winer, B. R. Yates, F. M. Addesa, P. Das, G. Dezoort, P. Elmer, A. Frankenthal, B. Greenberg, N. Haubrich, S. Higginbotham, A. Kalogeropoulos, G. Kopp, S. Kwan, D. Lange, D. Marlow, K. Mei, I. Ojalvo, J. Olsen, D. Stickland, C. Tully, S. Malik, S. Norberg, A. S. Bakshi, V. E. Barnes, R. Chawla, S. Das, L. Gutay, M. Jones, A. W. Jung, D. Kondratyev, A. M. Koshy, M. Liu, G. Negro, N. Neumeister, G. Paspalaki, S. Piperov, A. Purohit, J. F. Schulte, M. Stojanovic, J. Thieman, F. Wang, R. Xiao, W. Xie, J. Dolen, N. Parashar, D. Acosta, A. Baty, T. Carnahan, M. Decaro, S. Dildick, K. M. Ecklund, P. J. Fernández Manteca, S. Freed, P. Gardner, F. J. M. Geurts, A. Kumar, W. Li, B. P. Padley, R. Redjimi, J. Rotter, W. Shi, S. Yang, E. Yigitbasi, L. Zhang, Y. Zhang, A. Bodek, P. de Barbaro, R. Demina, J. L. Dulemba, C. Fallon, T. Ferbel, M. Galanti, A. Garcia-Bellido, O. Hindrichs, A. Khukhunaishvili, E. Ranken, R. Taus, G. P. Van Onsem, K. Goulianos, B. Chiarito, J. P. Chou, Y. Gershtein, E. Halkiadakis, A. Hart, M. Heindl, D. Jaroslawski, O. Karacheban, I. Laflotte, A. Lath, R. Montalvo, K. Nash, M. Osherson, H. Routray, S. Salur, S. Schnetzer, S. Somalwar, R. Stone, S. A. Thayil, S. Thomas, H. Wang, H. Acharya, A. G. Delannoy, S. Fiorendi, T. Holmes, E. Nibigira, S. Spanier, O. Bouhali, M. Dalchenko, A. Delgado, R. Eusebi, J. Gilmore, T. Huang, T. Kamon, H. Kim, S. Luo, S. Malhotra, R. Mueller, D. Overton, D. Rathjens, A. Safonov, N. Akchurin, J. Damgov, V. Hegde, K. Lamichhane, S. W. Lee, T. Mengke, S. Muthumuni, T. Peltola, I. Volobouev, A. Whitbeck, E. Appelt, S. Greene, A. Gurrola, W. Johns, A. Melo, F. Romeo, P. Sheldon, S. Tuo, J. Velkovska, J. Viinikainen, B. Cardwell, B. Cox, G. Cummings, J. Hakala, R. Hirosky, A. Ledovskoy, A. Li, C. Neu, C. E. Perez Lara, B. Tannenwald, P. E. Karchin, N. Poudyal, S. Banerjee, K. Black, T. Bose, S. Dasu, I. De Bruyn, P. Everaerts, C. Galloni, H. He, M. Herndon, A. Herve, C. K. Koraka, A. Lanaro, A. Loeliger, R. Loveless, J. Madhusudanan Sreekala, A. Mallampalli, A. Mohammadi, S. Mondal, G. Parida, D. Pinna, A. Savin, V. Shang, V. Sharma, W. H. Smith, D. Teague, H. F. Tsoi, W. Vetens, S. Afanasiev, V. Andreev, Yu. Andreev, T. Aushev, M. Azarkin, A. Babaev, A. Belyaev, V. Blinov, E. Boos, V. Borshch, D. Budkouski, V. Bunichev, V. Chekhovsky, R. Chistov, A. Dermenev, T. Dimova, I. Dremin, M. Dubinin, L. Dudko, V. Epshteyn, G. Gavrilov, V. Gavrilov, S. Gninenko, V. Golovtcov, N. Golubev, I. Golutvin, I. Gorbunov, V. Ivanchenko, Y. Ivanov, V. Kachanov, L. Kardapoltsev, V. Karjavine, A. Karneyeu, V. Kim, M. Kirakosyan, D. Kirpichnikov, M. Kirsanov, V. Klyukhin, O. Kodolova, D. Konstantinov, V. Korenkov, A. Kozyrev, N. Krasnikov, E. Kuznetsova, A. Lanev, P. Levchenko, A. Litomin, N. Lychkovskaya, V. Makarenko, A. Malakhov, V. Matveev, V. Murzin, A. Nikitenko, S. Obraztsov, A. Oskin, I. Ovtin, V. Palichik, P. Parygin, V. Perelygin, M. Perfilov, S. Petrushanko, S. Polikarpov, V. Popov, E. Popova, O. Radchenko, M. Savina, V. Savrin, D. Selivanova, V. Shalaev, S. Shmatov, S. Shulha, Y. Skovpen, S. Slabospitskii, V. Smirnov, D. Sosnov, V. Sulimov, E. Tcherniaev, A. Terkulov, O. Teryaev, I. Tlisova, M. Toms, A. Toropin, L. Uvarov, A. Uzunian, E. Vlasov, P. Volkov, A. Vorobyev, N. Voytishin, B. S. Yuldashev, A. Zarubin, I. Zhizhin, A. Zhokin, CMS Collaboration

**Affiliations:** 1 Yerevan Physics Institute, Yerevan, Armenia; 2 Institut für Hochenergiephysik, Vienna, Austria; 3 Universiteit Antwerpen, Antwerpen, Belgium; 4 Vrije Universiteit Brussel, Brussel, Belgium; 5 Université Libre de Bruxelles, Bruxelles, Belgium; 6 Ghent University, Ghent, Belgium; 7 Université Catholique de Louvain, Louvain-la-Neuve, Belgium; 8 Centro Brasileiro de Pesquisas Fisicas, Rio de Janeiro, Brazil; 9 Universidade do Estado do Rio de Janeiro, Rio de Janeiro, Brazil; 10 Universidade Estadual Paulista, Universidade Federal do ABC, São Paulo, Brazil; 11 Institute for Nuclear Research and Nuclear Energy, Bulgarian Academy of Sciences, Sofia, Bulgaria; 12 University of Sofia, Sofia, Bulgaria; 13 Instituto De Alta Investigación, Universidad de Tarapacá, Casilla 7 D, Arica, Chile; 14 Beihang University, Beijing, China; 15 Department of Physics, Tsinghua University, Beijing, China; 16 Institute of High Energy Physics, Beijing, China; 17 State Key Laboratory of Nuclear Physics and Technology, Peking University, Beijing, China; 18 Sun Yat-Sen University, Guangzhou, China; 19 Institute of Modern Physics and Key Laboratory of Nuclear Physics and Ion-beam Application (MOE)-Fudan University, Shanghai, China; 20 Zhejiang University, Hangzhou, Zhejiang, China; 21 Universidad de Los Andes, Bogota, Colombia; 22 Universidad de Antioquia, Medellin, Colombia; 23 University of Split, Faculty of Electrical Engineering, Mechanical Engineering and Naval Architecture, Split, Croatia; 24 Faculty of Science, University of Split, Split, Croatia; 25 Institute Rudjer Boskovic, Zagreb, Croatia; 26 University of Cyprus, Nicosia, Cyprus; 27 Charles University, Prague, Czech Republic; 28 Escuela Politecnica Nacional, Quito, Ecuador; 29 Universidad San Francisco de Quito, Quito, Ecuador; 30 Academy of Scientific Research and Technology of the Arab Republic of Egypt, Egyptian Network of High Energy Physics, Cairo, Egypt; 31 Center for High Energy Physics (CHEP-FU), Fayoum University, El-Fayoum, Egypt; 32 National Institute of Chemical Physics and Biophysics, Tallinn, Estonia; 33 Department of Physics, University of Helsinki, Helsinki, Finland; 34 Helsinki Institute of Physics, Helsinki, Finland; 35 Lappeenranta-Lahti University of Technology, Lappeenranta, Finland; 36 IRFU, CEA, Université Paris-Saclay, Gif-sur-Yvette, France; 37 Laboratoire Leprince-Ringuet, CNRS/IN2P3, Ecole Polytechnique, Institut Polytechnique de Paris, Palaiseau, France; 38 Université de Strasbourg, CNRS, IPHC UMR 7178, Strasbourg, France; 39 Institut de Physique des 2 Infinis de Lyon (IP2I ), Villeurbanne, France; 40 Georgian Technical University, Tbilisi, Georgia; 41 I. Physikalisches Institut, RWTH Aachen University, Aachen, Germany; 42 III. Physikalisches Institut A, RWTH Aachen University, Aachen, Germany; 43 III. Physikalisches Institut B, RWTH Aachen University, Aachen, Germany; 44 Deutsches Elektronen-Synchrotron, Hamburg, Germany; 45 University of Hamburg, Hamburg, Germany; 46 Karlsruher Institut fuer Technologie, Karlsruhe, Germany; 47 Institute of Nuclear and Particle Physics (INPP), NCSR Demokritos, Aghia Paraskevi, Greece; 48 National and Kapodistrian University of Athens, Athens, Greece; 49 National Technical University of Athens, Athens, Greece; 50 University of Ioánnina, Ioannina, Greece; 51 MTA-ELTE Lendület CMS Particle and Nuclear Physics Group, Eötvös Loránd University, Budapest, Hungary; 52 Wigner Research Centre for Physics, Budapest, Hungary; 53 Institute of Nuclear Research ATOMKI, Debrecen, Hungary; 54 Institute of Physics, University of Debrecen, Debrecen, Hungary; 55 Karoly Robert Campus, MATE Institute of Technology, Gyongyos, Hungary; 56 Panjab University, Chandigarh, India; 57 University of Delhi, Delhi, India; 58 Saha Institute of Nuclear Physics, HBNI, Kolkata, India; 59 Indian Institute of Technology Madras, Madras, India; 60 Bhabha Atomic Research Centre, Mumbai, India; 61 Tata Institute of Fundamental Research-A, Mumbai, India; 62 Tata Institute of Fundamental Research-B, Mumbai, India; 63 National Institute of Science Education and Research, An OCC of Homi Bhabha National Institute, Bhubaneswar, Odisha India; 64 Indian Institute of Science Education and Research (IISER), Pune, India; 65 Isfahan University of Technology, Isfahan, Iran; 66 Institute for Research in Fundamental Sciences (IPM), Tehran, Iran; 67 University College Dublin, Dublin, Ireland; 68 INFN Sezione di Bari, Università di Bari, Politecnico di Bari, Bari, Italy; 69 INFN Sezione di Bologna, Università di Bologna, Bologna, Italy; 70 INFN Sezione di Catania, Università di Catania, Catania, Italy; 71 INFN Sezione di Firenze, Università di Firenze, Firenze, Italy; 72 INFN Laboratori Nazionali di Frascati, Frascati, Italy; 73 INFN Sezione di Genova, Università di Genova, Genoa, Italy; 74 INFN Sezione di Milano-Bicocca, Università di Milano-Bicocca, Milan, Italy; 75 INFN Sezione di Napoli, Università di Napoli ’Federico II’, Napoli, Italy; Università della Basilicata, Potenza, Italy; Università G. Marconi, Rome, Italy; 76 INFN Sezione di Padova, Università di Padova, Padova, Italy; Università di Trento, Trento, Italy; 77 INFN Sezione di Pavia, Università di Pavia, Pavia, Italy; 78 INFN Sezione di Perugia, Università di Perugia, Perugia, Italy; 79 INFN Sezione di Pisa, Università di Pisa, Scuola Normale Superiore di Pisa, Pisa, Italy; Università di Siena, Siena, Italy; 80 INFN Sezione di Roma, Sapienza Università di Roma, Rome, Italy; 81 INFN Sezione di Torino, Università di Torino, Torino, Italy; Università del Piemonte Orientale, Novara, Italy; 82 INFN Sezione di Trieste, Università di Trieste, Trieste, Italy; 83 Kyungpook National University, Daegu, Korea; 84 Chonnam National University, Institute for Universe and Elementary Particles, Kwangju, Korea; 85 Hanyang University, Seoul, Korea; 86 Korea University, Seoul, Korea; 87 Kyung Hee University, Department of Physics, Seoul, Korea; 88 Sejong University, Seoul, Korea; 89 Seoul National University, Seoul, Korea; 90 University of Seoul, Seoul, Korea; 91 Yonsei University, Department of Physics, Seoul, Korea; 92 Sungkyunkwan University, Suwon, Korea; 93 College of Engineering and Technology, American University of the Middle East (AUM), Dasman, Kuwait; 94 Riga Technical University, Riga, Latvia; 95 Vilnius University, Vilnius, Lithuania; 96 National Centre for Particle Physics, Universiti Malaya, Kuala Lumpur, Malaysia; 97 Universidad de Sonora (UNISON), Hermosillo, Mexico; 98 Centro de Investigacion y de Estudios Avanzados del IPN, Mexico City, Mexico; 99 Universidad Iberoamericana, Mexico City, Mexico; 100 Benemerita Universidad Autonoma de Puebla, Puebla, Mexico; 101 University of Montenegro, Podgorica, Montenegro; 102 National Centre for Physics, Quaid-I-Azam University, Islamabad, Pakistan; 103 AGH University of Science and Technology Faculty of Computer Science, Electronics and Telecommunications, Kraków, Poland; 104 National Centre for Nuclear Research, Swierk, Poland; 105 Institute of Experimental Physics, Faculty of Physics, University of Warsaw, Warsaw, Poland; 106 Laboratório de Instrumentação e Física Experimental de Partículas, Lisbon, Portugal; 107 VINCA Institute of Nuclear Sciences, University of Belgrade, Belgrade, Serbia; 108 Centro de Investigaciones Energéticas Medioambientales y Tecnológicas (CIEMAT), Madrid, Spain; 109 Universidad Autónoma de Madrid, Madrid, Spain; 110 Universidad de Oviedo, Instituto Universitario de Ciencias y Tecnologías Espaciales de Asturias (ICTEA), Oviedo, Spain; 111 Instituto de Física de Cantabria (IFCA), CSIC-Universidad de Cantabria, Santander, Spain; 112 University of Colombo, Colombo, Sri Lanka; 113 University of Ruhuna, Department of Physics, Matara, Sri Lanka; 114 CERN, European Organization for Nuclear Research, Geneva, Switzerland; 115 Paul Scherrer Institut, Villigen, Switzerland; 116 ETH Zurich-Institute for Particle Physics and Astrophysics (IPA), Zurich, Switzerland; 117 Universität Zürich, Zurich, Switzerland; 118 National Central University, Chung-Li, Taiwan; 119 National Taiwan University (NTU), Taipei, Taiwan; 120 Chulalongkorn University, Faculty of Science, Department of Physics, Bangkok, Thailand; 121 Çukurova University, Physics Department, Science and Art Faculty, Adana, Turkey; 122 Middle East Technical University, Physics Department, Ankara, Turkey; 123 Bogazici University, Istanbul, Turkey; 124 Istanbul Technical University, Istanbul, Turkey; 125 Istanbul University, Istanbul, Turkey; 126 Institute for Scintillation Materials of National Academy of Science of Ukraine, Kharkiv, Ukraine; 127 National Science Centre, Kharkiv Institute of Physics and Technology, Kharkiv, Ukraine; 128 University of Bristol, Bristol, UK; 129 Rutherford Appleton Laboratory, Didcot, UK; 130 Imperial College, London, UK; 131 Brunel University, Uxbridge, UK; 132 Baylor University, Waco, TX USA; 133 Catholic University of America, Washington, DC USA; 134 The University of Alabama, Tuscaloosa, AL USA; 135 Boston University, Boston, MA USA; 136 Brown University, Providence, RI USA; 137 University of California, Davis, Davis, CA USA; 138 University of California, Los Angeles, CA USA; 139 University of California, Riverside, Riverside, CA USA; 140 University of California, San Diego, La Jolla, CA USA; 141 Department of Physics, University of California, Santa Barbara, Santa Barbara, CA USA; 142 California Institute of Technology, Pasadena, CA USA; 143 Carnegie Mellon University, Pittsburgh, PA USA; 144 University of Colorado Boulder, Boulder, CO USA; 145 Cornell University, Ithaca, NY USA; 146 Fermi National Accelerator Laboratory, Batavia, IL USA; 147 University of Florida, Gainesville, FL USA; 148 Florida State University, Tallahassee, FL USA; 149 Florida Institute of Technology, Melbourne, FL USA; 150 University of Illinois at Chicago (UIC), Chicago, IL USA; 151 The University of Iowa, Iowa City, IA USA; 152 Johns Hopkins University, Baltimore, MD USA; 153 The University of Kansas, Lawrence, KS USA; 154 Kansas State University, Manhattan, KS USA; 155 Lawrence Livermore National Laboratory, Livermore, CA USA; 156 University of Maryland, College Park, MD USA; 157 Massachusetts Institute of Technology, Cambridge, MA USA; 158 University of Minnesota, Minneapolis, MN USA; 159 University of Mississippi, Oxford, MS USA; 160 University of Nebraska-Lincoln, Lincoln, NE USA; 161 State University of New York at Buffalo, Buffalo, NY USA; 162 Northeastern University, Boston, MA USA; 163 Northwestern University, Evanston, IL USA; 164 University of Notre Dame, Notre Dame, IN USA; 165 The Ohio State University, Columbus, OH USA; 166 Princeton University, Princeton, NJ USA; 167 University of Puerto Rico, Mayaguez, PR USA; 168 Purdue University, West Lafayette, IN USA; 169 Purdue University Northwest, Hammond, IN USA; 170 Rice University, Houston, TX USA; 171 University of Rochester, Rochester, NY USA; 172 The Rockefeller University, New York, NY USA; 173 Rutgers, The State University of New Jersey, Piscataway, NJ USA; 174 University of Tennessee, Knoxville, TN USA; 175 Texas A &M University, College Station, TX USA; 176 Texas Tech University, Lubbock, TX USA; 177 Vanderbilt University, Nashville, TN USA; 178 University of Virginia, Charlottesville, VA USA; 179 Wayne State University, Detroit, MI USA; 180 University of Wisconsin-Madison, Madison, WI USA; 181 Authors affiliated with an institute or an international laboratory covered by a cooperation agreement with CERN, Geneva, Switzerland; 182 Yerevan State University, Yerevan, Armenia; 183 TU Wien, Vienna, Austria; 184 Institute of Basic and Applied Sciences, Faculty of Engineering, Arab Academy for Science, Technology and Maritime Transport, Alexandria, Egypt; 185 Université Libre de Bruxelles, Bruxelles, Belgium; 186 Universidade Estadual de Campinas, Campinas, Brazil; 187 Federal University of Rio Grande do Sul, Porto Alegre, Brazil; 188 UFMS, Nova Andradina, Brazil; 189 The University of the State of Amazonas, Manaus, Brazil; 190 University of Chinese Academy of Sciences, Beijing, China; 191 Nanjing Normal University Department of Physics, Nanjing, China; 192 The University of Iowa, Iowa City, IA USA; 193 University of Chinese Academy of Sciences, Beijing, China; 194 an institute or an international laboratory covered by a cooperation agreement with CERN, Geneva, Switzerland; 195 Cairo University, Cairo, Egypt; 196 Suez University, Suez, Egypt; 197 British University in Egypt, Cairo, Egypt; 198 Purdue University, West Lafayette, IN USA; 199 Université de Haute Alsace, Mulhouse, France; 200 Department of Physics, Tsinghua University, Beijing, China; 201 Tbilisi State University, Tbilisi, Georgia; 202 Erzincan Binali Yildirim University, Erzincan, Turkey; 203 University of Hamburg, Hamburg, Germany; 204 RWTH Aachen University, III. Physikalisches Institut A, Aachen, Germany; 205 Isfahan University of Technology, Isfahan, Iran; 206 Bergische University Wuppertal (BUW), Wuppertal, Germany; 207 Brandenburg University of Technology, Cottbus, Germany; 208 Forschungszentrum Jülich, Juelich, Germany; 209 CERN, European Organization for Nuclear Research, Geneva, Switzerland; 210 Physics Department, Faculty of Science, Assiut University, Assiut, Egypt; 211 Karoly Robert Campus, MATE Institute of Technology, Gyongyos, Hungary; 212 Wigner Research Centre for Physics, Budapest, Hungary; 213 Institute of Physics, University of Debrecen, Debrecen, Hungary; 214 Institute of Nuclear Research ATOMKI, Debrecen, Hungary; 215 Universitatea Babes-Bolyai-Facultatea de Fizica, Cluj-Napoca, Romania; 216 Faculty of Informatics, University of Debrecen, Debrecen, Hungary; 217 Punjab Agricultural University, Ludhiana, India; 218 UPES-University of Petroleum and Energy Studies, Dehradun, India; 219 University of Visva-Bharati, Santiniketan, India; 220 University of Hyderabad, Hyderabad, India; 221 Indian Institute of Science (IISc), Bangalore, India; 222 Indian Institute of Technology (IIT), Mumbai, India; 223 IIT Bhubaneswar, Bhubaneswar, India; 224 Institute of Physics, Bhubaneswar, India; 225 Deutsches Elektronen-Synchrotron, Hamburg, Germany; 226 Sharif University of Technology, Tehran, Iran; 227 Department of Physics, University of Science and Technology of Mazandaran, Behshahr, Iran; 228 Helwan University, Cairo, Egypt; 229 Italian National Agency for New Technologies, Energy and Sustainable Economic Development, Bologna, Italy; 230 Centro Siciliano di Fisica Nucleare e di Struttura Della Materia, Catania, Italy; 231 Scuola Superiore Meridionale, Università di Napoli ’Federico II’, Naples, Italy; 232 Fermi National Accelerator Laboratory, Batavia, IL USA; 233 Università di Napoli ’Federico II’, Naples, Italy; 234 Ain Shams University, Cairo, Egypt; 235 Consiglio Nazionale delle Ricerche-Istituto Officina dei Materiali, Perugia, Italy; 236 Department of Applied Physics, Faculty of Science and Technology, Universiti Kebangsaan Malaysia, Bangi, Malaysia; 237 Consejo Nacional de Ciencia y Tecnología, Mexico City, Mexico; 238 IRFU, CEA, Université Paris-Saclay, Gif-sur-Yvette, France; 239 Faculty of Physics, University of Belgrade, Belgrade, Serbia; 240 Trincomalee Campus, Eastern University, Sri Lanka, Nilaveli, Sri Lanka; 241 INFN Sezione di Pavia, Università di Pavia, Pavia, Italy; 242 National and Kapodistrian University of Athens, Athens, Greece; 243 Ecole Polytechnique Fédérale Lausanne, Lausanne, Switzerland; 244 Universität Zürich, Zurich, Switzerland; 245 Stefan Meyer Institute for Subatomic Physics, Vienna, Austria; 246 Laboratoire d’Annecy-le-Vieux de Physique des Particules, IN2P3-CNRS, Annecy-le-Vieux, France; 247 Near East University, Research Center of Experimental Health Science, Mersin, Turkey; 248 Konya Technical University, Konya, Turkey; 249 Izmir Bakircay University, Izmir, Turkey; 250 Adiyaman University, Adiyaman, Turkey; 251 Istanbul Gedik University, Istanbul, Turkey; 252 Necmettin Erbakan University, Konya, Turkey; 253 Bozok Universitetesi Rektörlügü, Yozgat, Turkey; 254 Marmara University, Istanbul, Turkey; 255 Milli Savunma University, Istanbul, Turkey; 256 Kafkas University, Kars, Turkey; 257 Istanbul University-Cerrahpasa, Faculty of Engineering, Istanbul, Turkey; 258 Yildiz Technical University, Istanbul, Turkey; 259 Vrije Universiteit Brussel, Brussel, Belgium; 260 School of Physics and Astronomy, University of Southampton, Southampton, UK; 261 University of Bristol, Bristol, UK; 262 IPPP Durham University, Durham, UK; 263 Monash University, Faculty of Science, Clayton, Australia; 264 Università di Torino, Turin, Italy; 265 Bethel University, St. Paul, MN USA; 266 Karamanoğlu Mehmetbey University, Karaman, Turkey; 267 California Institute of Technology, Pasadena, CA USA; 268 United States Naval Academy, Annapolis, MD USA; 269 Bingol University, Bingol, Turkey; 270 Georgian Technical University, Tbilisi, Georgia; 271 Sinop University, Sinop, Turkey; 272 Erciyes University, Kayseri, Turkey; 273 Institute of Modern Physics and Key Laboratory of Nuclear Physics and Ion-beam Application (MOE)-Fudan University, Shanghai, China; 274 Texas A &M University at Qatar, Doha, Qatar; 275 Kyungpook National University, Daegu, Korea; 276 another institute or international laboratory covered by a cooperation agreement with CERN, Geneva, Switzerland; 277 Yerevan Physics Institute, Yerevan, Armenia; 278 University of Florida, Gainesville, FL USA; 279 Imperial College, London, UK; 280 Institute of Nuclear Physics of the Uzbekistan Academy of Sciences, Tashkent, Uzbekistan; 281 CERN, 1211 Geneva 23, Switzerland

## Abstract

A measurement of the jet mass distribution in hadronic decays of Lorentz-boosted top quarks is presented. The measurement is performed in the lepton + jets channel of top quark pair production ($\hbox {t}\overline{\hbox {t}}$) events, where the lepton is an electron or muon. The products of the hadronic top quark decay are reconstructed using a single large-radius jet with transverse momentum greater than 400$\,\text {Ge}\hspace{-.08em}\text {V}$. The data were collected with the CMS detector at the LHC in proton-proton collisions and correspond to an integrated luminosity of 138$\,\text {fb}^{-1}$. The differential $\hbox {t}\overline{\hbox {t}}$ production cross section as a function of the jet mass is unfolded to the particle level and is used to extract the top quark mass. The jet mass scale is calibrated using the hadronic W boson decay within the large-radius jet. The uncertainties in the modelling of the final state radiation are reduced by studying angular correlations in the jet substructure. These developments lead to a significant increase in precision, and a top quark mass of $173.06 \pm 0.84\,\text {Ge}\hspace{-.08em}\text {V}$.

## Introduction

The top quark is the most massive elementary particle discovered so far [1, 2]. Because of its high mass $m_{\textrm{t}}$ and its large Yukawa coupling it plays a crucial role in the electroweak sector of the standard model (SM) of particle physics. Precise measurements of $m_{\textrm{t}}$ allow for stringent tests of the validity of the SM [3–5] and place constraints on the stability of the electroweak vacuum [6–8].

Direct measurements of $m_{\textrm{t}}$ using the top quark decay products have already achieved a precision of about 0.5$\,\text {Ge}\hspace{-.08em}\text {V}$ [9–15]. In these measurements, observables with high sensitivity to the value of $m_{\textrm{t}}$ are constructed. Measured distributions in these observables are compared to detector level simulations to extract the value of $m_{\textrm{t}}$ that fits the data best. The predictions rely on a precise modelling of the parton shower and hadronisation process, which cannot be calculated from first principles, and are thus subject to corresponding systematic uncertainties. In addition, uncertainties of the size 0.5–1$\,\text {Ge}\hspace{-.08em}\text {V}$ exist in the translation of $m_{\textrm{t}}$ extracted from event generators to a value of $m_{\textrm{t}}$ in a well-defined renormalisation scheme [16, 17], as used in precise analytic calculations in quantum field theory.

A different approach is the determination of $m_{\textrm{t}}$ from cross section measurements corrected for detector effects. To facilitate a direct comparison to analytic calculations from first principles, these measurements have to be corrected to the parton level, which represents the $\hbox {t}\overline{\hbox {t}}$ pair before its decay. The corrections applied need to include effects from the top quark decay and the hadronisation of its colour-charged decay products. The inclusive cross section of top quark pair ($\hbox {t}\overline{\hbox {t}}$) production can be measured precisely and has been used to extract a value of the top quark pole mass by a comparison to fixed-order calculations in perturbative quantum chromodynamics (QCD). Such measurements have been carried out by the D0 [18, 19], ATLAS [20–22], and CMS [23–25] Collaborations. These measurements of the total $\hbox {t}\overline{\hbox {t}}$ cross section are sensitive to various sources of uncertainties, which can not be constrained in situ during the extraction of $m_{\textrm{t}}$, resulting in a precision of about 2$\,\text {Ge}\hspace{-.08em}\text {V}$. Differential cross section measurements can also be used for measuring $m_{\textrm{t}}$ [26–29]. A multi-differential cross section measurement has been performed by the CMS Collaboration, achieving an uncertainty of 0.8$\,\text {Ge}\hspace{-.08em}\text {V}$ in the top quark pole mass [30]. The shape of the measured distributions close to the $\hbox {t}\overline{\hbox {t}}$ production threshold is sensitive to the value of $m_{\textrm{t}}$, and a more precise result is achieved compared to the inclusive cross section measurements.

An alternative method which combines the advantages of the two approaches is the determination of $m_{\textrm{t}}$ from a measurement of the jet mass $m_{\textrm{jet}}$ in events with Lorentz-boosted top quarks [31–33]. At high energies, the decay products of top quarks are Lorentz boosted and merge into a single large-radius jet. The peak position of the distribution in $m_{\textrm{jet}}$ is sensitive to $m_{\textrm{t}}$ and allows for a precise measurement of $m_{\textrm{t}}$ [34]. The unfolding of the data to the level of stable particles will allow for a comparison to analytic calculations in perturbative QCD, once these become available. This enables a measurement of the top quark pole mass from the shape of a distribution at the particle level. Presently, analytic calculations for $m_{\textrm{jet}}$ are restricted to top quark transverse momenta $p_{\textrm{T}} >750\,\text {Ge}\hspace{-.08em}\text {V}$ [34], a requirement which results in too few events in data for a differential cross section measurement using the current CERN LHC data sets. Previous measurements by the CMS Collaboration using proton-proton (${\text {p}} {\text {p}}$) collision data at $\sqrt{s}=8\,\text {Te}\hspace{-.08em}\text {V}$ [35] and 13$\,\text {Te}\hspace{-.08em}\text {V}$ [36] with a top quark $p_{\textrm{T}} >400\,\text {Ge}\hspace{-.08em}\text {V}$, have reached an uncertainty of 2.5$\,\text {Ge}\hspace{-.08em}\text {V}$, where $m_{\textrm{t}}$ has been determined using event generators. The results are compatible with those obtained from $\hbox {t}\overline{\hbox {t}}$ production at lower energy scales. In this article, we present a measurement of the differential cross section for $\hbox {t}\overline{\hbox {t}}$ production as a function of the large-radius jet mass with significantly improved statistical and systematic uncertainties. The measurement is used to determine $m_{\textrm{t}}$ using event generators at next-to-leading order (NLO) precision in QCD. The approach is complementary to measurements close to threshold production with fully resolved final state objects. This provides a precise test of the validity of the approximations made in event generators and the corresponding systematic uncertainties.

In the lepton+jets channel of $\hbox {t}\overline{\hbox {t}}$ production, the final state is obtained from one top quark decaying to a b quark and leptons, $\textrm{t} \rightarrow {\text {b}} {\text {W}} \rightarrow {\text {b}} {\ell } {{\upnu }} _{\!\ell }$, and the second decaying hadronically, . Here, the term lepton denotes an electron or muon. This final state combines the advantages of a clear signature from the leptonic W boson decay, with a small background from events with jets from light-flavour quarks and gluons. The large $\hbox {t}\overline{\hbox {t}}$ branching fraction for the lepton+jets channel also results in large event samples. In addition, in case of $\hbox {t}\overline{\hbox {t}}$ production with high top quark $p_{\textrm{T}}$, the hadronic decay allows the full reconstruction of the top quark decay within a single large-radius jet with $p_{\textrm{T}} >400\,\text {Ge}\hspace{-.08em}\text {V}$, provided that the decay products are produced within the detector acceptance. The lepton serves as a means to select $\hbox {t}\overline{\hbox {t}}$ events, and the mass of the large-radius jet in the opposite hemisphere of the event is the measurable for this analysis. The lepton is not necessarily isolated, because the large Lorentz boost can result in particles from the fragmentation of the b quark to be produced inside of the isolation cone around the lepton. The analysis strategy follows the one from the previous measurement at 13$\,\text {Te}\hspace{-.08em}\text {V}$ [36].

In this article, we analyse 13$\,\text {Te}\hspace{-.08em}\text {V}$ ${\text {p}} {\text {p}}$ collision data, recorded in the years 2016 to 2018 and corresponding to an integrated luminosity of 138$\,\text {fb}^{-1}$. Besides the improved statistical precision, the leading systematic uncertainties are reduced by using a dedicated calibration of the jet mass scale (JMS) and a detailed study of the effects from final state radiation (FSR) in large-radius jets.

In the previous measurements of $m_{\textrm{jet}}$ in boosted $\hbox {t}\overline{\hbox {t}}$ events [35, 36], the uncertainties in the jet energy scale (JES) have been propagated to $m_{\textrm{jet}}$. For these the JES uncertainties are the leading experimental systematic uncertainties. While the JES, and therefore the jet momentum, can be determined precisely using the $p_{\textrm{T}}$ balance or the MPF (missing transverse momentum projection fraction) methods [37, 38], these methods do not necessarily provide the most precise calibrations for $m_{\textrm{jet}}$. In this article, we calibrate the JMS by reconstructing the W boson mass from two subjets within the large-radius jet. A fit to data in the peak region of the jet mass results in a JMS with smaller uncertainties.

The FSR is modelled by the parton showers in the event generators, which are matched to the simulation of the hard process. The value of the strong coupling used in the FSR shower, evaluated at the mass of the Z boson, $\alpha _{\textrm{S}} ^{{\textrm{FSR}}}(m_{{\hbox {Z}}}^{2})$, is an important parameter that affects the amount of FSR. Changes in its value can cause large differences in the substructure of large-radius jets. Observables probing the angular distributions of the energy density within a jet, such as *N*-subjettiness [39, 40] ratios, are very sensitive to the amount of FSR in the simulation. In this article, we measure distributions in *N*-subjettiness ratios calculated for large-radius jets, and use these to constrain the value of $\alpha _{\textrm{S}} ^{{\textrm{FSR}}}(m_{{\hbox {Z}}}^{2})$ used in the modelling of FSR. This leads to smaller uncertainties in $m_{\textrm{jet}}$ from the FSR modelling compared to the usual variations of the scale $\mu$ in $\alpha _{\textrm{S}} ^{{\textrm{FSR}}}(\mu ^{2})$ [36, 41, 42].

Tabulated results are provided in the HEPData record for this analysis [43].

## The CMS detector

The central feature of the CMS detector is a superconducting solenoid of 6$\,\text {m}$ internal diameter, providing a magnetic field of 3.8$\,\text {T}$. A silicon pixel and strip tracker, a lead tungstate crystal electromagnetic calorimeter (ECAL), and a brass and scintillator hadron calorimeter (HCAL), each composed of a central barrel and two endcap sections, reside within the solenoid volume. Forward calorimeters extend the pseudorapidity ($\eta$) coverage provided by the barrel and endcap detectors. Muons are detected in gas-ionisation chambers embedded in the steel flux-return yoke outside the solenoid. A more detailed description of the CMS detector, together with a definition of the coordinate system, can be found in Ref. [44]. Between the 2016 and 2017 data taking runs, the CMS pixel detector was upgraded with additional layers in the barrel and endcap regions of the CMS detector. Details about the changes can be found in Ref. [45].

Events of interest are selected using a two-tiered trigger system. The first level, composed of custom hardware processors, uses information from the calorimeters and muon detectors to select events at a rate of around 100$\,\text {kHz}$ within a fixed latency of about 4$\,\upmu \text {s}$ [46]. The second level, known as the high-level trigger, consists of a farm of processors running a version of the full event reconstruction software optimised for fast processing, and reduces the event rate to around 1$\,\text {kHz}$ before data storage [47].

## Data and simulated samples

The measurement is performed in the lepton+jets final state of $\hbox {t}\overline{\hbox {t}}$ production. The event selection is based on the presence of a single lepton which uses the data selected by single-lepton triggers [46, 47]. Muon candidates are required to have $p_{\textrm{T}} >50\,\text {Ge}\hspace{-.08em}\text {V}$ and $|\eta |<2.4$, without any requirement on the isolation of the muon. In the electron channel, we use a combination of triggers. The first trigger requires electron candidates with $|\eta |<2.5$ that are isolated and have a minimum $p_{\textrm{T}}$ of 27, 35, or 32$\,\text {Ge}\hspace{-.08em}\text {V}$ for the years 2016, 2017, and 2018, respectively. A second trigger selects electron candidates with $p_{\textrm{T}} > 120\,\text {Ge}\hspace{-.08em}\text {V}$, without an isolation requirement. In addition a single-photon trigger is used for selecting electrons without a track requirement. This trigger selects photon candidates with a minimum $p_{\textrm{T}}$ of 175$\,\text {Ge}\hspace{-.08em}\text {V}$ in 2016, and 200$\,\text {Ge}\hspace{-.08em}\text {V}$ in 2017 and 2018. The photon trigger ensures a stable selection efficiency for electrons at high $p_{\textrm{T}}$ because selection criteria applied to clusters in the ECAL are less strict than those used by the electron trigger. In the offline analysis, muons and electrons are selected with $|\eta |<2.4$ and $p_{\textrm{T}} >55\,\text {Ge}\hspace{-.08em}\text {V}$, ensuring that selected events are in the plateau region of the trigger efficiency. After this selection, the average efficiency of the muon trigger is 91, 90, and 91% for 2016, 2017, and 2018, respectively. The combination of the three electron and photon triggers provides high efficiency over the full range in $p_{\textrm{T}}$ considered in this analysis, which is comparable to that obtained using the muon triggers. For lepton $p_{\textrm{T}} <120\,\text {Ge}\hspace{-.08em}\text {V}$, the top quark decay is less collimated and the b jet does not overlap with the lepton isolation cone. In this case, the event selection efficiency is greater than 90% for triggers with an isolation requirement. For $p_{\textrm{T}} >120\,\text {Ge}\hspace{-.08em}\text {V}$, the nonisolated electron trigger has an average efficiency of 95%, increasing to nearly 100% for $p_{\textrm{T}} >200\,\text {Ge}\hspace{-.08em}\text {V}$, where the high $p_{\textrm{T}}$ efficiency is calculated in combination with the photon trigger. The total data set corresponds to an integrated luminosity of 138$\,\text {fb}^{-1}$, with 36.3$\,\text {fb}^{-1}$ [48], 41.5$\,\text {fb}^{-1}$ [49], and 59.7$\,\text {fb}^{-1}$ [50] recorded in the years 2016, 2017, and 2018, respectively.

For each of the three years of data taking, the processes relevant for this analysis are simulated individually using a Monte Carlo (MC) simulation technique and they are normalised to the integrated luminosity of each year. The $\hbox {t}\overline{\hbox {t}}$ process is simulated at NLO using the powheg  v2 [51–56] generator with a top quark mass of 172.5$\,\text {Ge}\hspace{-.08em}\text {V}$. We adjust the total cross section to 831.8$\,\text {pb}$, obtained from a prediction at next-to-NLO (NNLO) precision in QCD, including resummation of next-to-next-to-leading logarithmic soft gluon terms, using the computer program Top++  2.0 [57]. We simulate additional $\hbox {t}\overline{\hbox {t}}$ samples with $m_{\textrm{t}} =169.5$, 171.5, 173.5, and 175.5$\,\text {Ge}\hspace{-.08em}\text {V}$, which are used for studying the dependence of the measured cross section on the value of $m_{\textrm{t}}$ used in simulation, and for the extraction of $m_{\textrm{t}}$. The background contribution from electroweak single $\textrm{t}$ production is generated at NLO using powheg, and the background is generated at leading order (LO) using MadGraph 5_amc@nlo v2.2.2 [58, 59]. The cross section for single $\textrm{t}$ production in association with a W boson is adjusted to approximate NNLO calculations taken from Refs. [60, 61]. The single top quark *s*- and *t*-channel cross sections are adjusted to predictions at NLO precision obtained with hathor v2.1 [62]. Events from Drell–Yan (DY) production with additional jets are simulated at LO using MadGraph 5_amc@nlo and normalised to the NLO cross section [63]. The production of two heavy gauge bosons with additional jets, and events in which jets are produced only through QCD interactions are simulated at LO using the pythia event generator in version 8.212 [64] for the simulation of 2016 data and version 8.230 for 2017 and 2018. The diboson and QCD multijet samples are referred to as “other SM” backgrounds in the following. The NNPDF3.0 [65] parton distribution functions (PDFs) are used for 2016 simulations and the NNPDF3.1 [66] PDFs are used for 2017 and 2018 simulations.

In all processes, the hadronisation, parton showers, and multiple parton interactions are simulated with pythia. In samples simulated with MadGraph 5_amc@nlo, the matrix element calculation is matched to the parton showers using the FxFx [67] and MLM [68] algorithms for NLO and LO, respectively. In the simulation of 2016 data, pythia  8.212 is used with the underlying event (UE) tune CUETP8M2T4 [69] for the simulation of $\hbox {t}\overline{\hbox {t}}$ and single top quark production in the *t* channel. In this tune, $\alpha _{\textrm{S}} ^{{\textrm{FSR}}}(m_{{\hbox {Z}}}^{2}) = 0.1365$ is used for the simulation of FSR. All other simulated samples in 2016 use the CUETP8M1 [41, 70] tune. For the 2017 and 2018 data, pythia  8.230 is used with the CP5 [69] tune. Here, a value of $\alpha _{\textrm{S}} ^{{\textrm{FSR}}}(m_{{\hbox {Z}}}^{2}) = 0.118$ is used. The detector response is simulated with the Geant4 package [71, 72].

Additional inelastic ${\text {p}} {\text {p}}$ collision events are simulated using pythia and superimposed on simulated events to model the effect of additional ${\text {p}} {\text {p}}$ collisions within the same or adjacent bunch crossings (pileup). We use a total inelastic cross section of 69.2$\,\text {mb}$ [73] to estimate the expected number of ${\text {p}} {\text {p}}$ interactions per bunch crossing and correct the simulation to match the corresponding distribution to that observed in data.

## Event reconstruction

The particle-flow (PF) algorithm [74] aims to reconstruct and identify each individual particle in an event, using an optimised combination of information from the various elements of the CMS detector. The candidate vertex with the largest sum of the square of the transverse momenta $p_{\textrm{T}} ^2$ of the physics objects is taken to be the leading primary ${\text {p}} {\text {p}}$ interaction vertex. The physics objects are the jets, clustered using the anti-$k_{\textrm{T}}$ jet finding algorithm [75, 76] with a distance parameter of $R=0.4$ with tracks assigned to candidate vertices as inputs, and the associated missing transverse momentum, taken as the negative vector sum of the $p_{\textrm{T}}$ of those jets. More details are given in Section 9.4.1 of Ref. [77].

Muons are reconstructed from tracks in the inner tracker and hits in the muon system using the PF algorithm. The muon momentum is obtained from the curvature of the corresponding track [78]. For electron reconstruction, clusters in the ECAL are connected to tracks in the inner tracker. The electron energy is determined by a combination of the electron momentum at the primary interaction vertex as determined by the tracker, the energy of the corresponding cluster in the ECAL, and the sum of all bremsstrahlung photons spatially compatible with originating from the electron track [79]. The energy of photons is directly obtained from the ECAL measurement [79]. Both muons and electrons have to pass tight quality criteria developed by the CMS Collaboration to ensure a proper reconstruction [78, 79]. The energy of charged hadrons is determined from a combination of their momentum measured in the tracker and the matching ECAL and HCAL energy deposits. Finally, the energy of neutral hadrons is obtained from the corresponding corrected ECAL and HCAL energy [74].

Jets are reconstructed from PF candidates using the anti-$k_{\textrm{T}}$ [75] or the XCone (eXclusive Cone) [80] algorithm as implemented in the FastJet software package [76]. Two sets of anti-$k_{\textrm{T}}$ jets are obtained using distance parameters of $R=0.4$ (AK4 jets) and 0.8 (AK8 jets). In the jet clustering procedure, charged PF candidates are excluded if they are associated with pileup vertices. While AK4 jets are used mostly for the identification of b jets in this analysis, AK8 jets are used to study the influence of FSR on the jet substructure as described in Sect. 8. For the XCone jets, a specialized two-step clustering procedure [81] is used. Being an exclusive algorithm, XCone always returns a requested number of jets. This feature of the algorithm can be leveraged to efficiently reconstruct the boosted $\hbox {t}\overline{\hbox {t}}$ final state. At first, the XCone algorithm is run finding exactly two large-radius jets with a distance parameter of $R=1.2$. This step takes all PF candidates, after removing charged particles assigned to a pileup vertex, as an input and aims to reconstruct the two top quark decays of the $\hbox {t}\overline{\hbox {t}}$ process in separate jets. As a second step, all PF candidates clustered into a large-radius XCone jet are input to the XCone algorithm again, which is now required to find three XCone subjets, $N_{\text {sub}}=3$, with a distance parameter $R_{\text {sub}}=0.4$. The second step aims to reconstruct the three-prong decay  while minimising the effects of uncorrelated soft radiation or additional energy deposits from pileup. The final XCone jets are then defined as the sum of the four-momenta of their respective subjets. In this way, all particles not clustered into the three subjets are removed from the large-radius XCone jets, similar to the trimming algorithm [82]. The jet mass is calculated from the sum of the four-momenta of all particles clustered into the subjets. Since no lepton selection has been applied at this stage, the XCone reconstruction will also reconstruct $\textrm{t} \rightarrow {\text {b}} {\text {W}} \rightarrow {\text {b}} {\ell } {{\upnu }} _{\!\ell }$ with three subjets. We have verified that the difference from the more natural choice of two XCone subjets for the reconstruction of the leptonic decay does not significantly affect the identification of the leptonic XCone jet and the event reconstruction. The XCone jet with larger angular distance to the identified single lepton is selected as the measurement jet and is labelled “XCone jet” in the following. The XCone jet closer to the lepton is referred to as “second XCone jet”. Here, the angular distance between two objects is defined as ${\varDelta }R = {\tiny {\sqrt{\smash [b]{({\varDelta }\eta )^2+({\varDelta }\phi )^2}}}^{}}$, where $\phi$ is the azimuthal angle in radians. The four-momenta of identified leptons are subtracted from AK4 jets and XCone subjets if they are within ${\varDelta }R<0.4$ of the respective (sub)jet.

Jet energy corrections (JECs) [38] derived for AK4 jets are applied to AK4 jets, as well as to XCone subjets in this analysis. These JECs include corrections for contributions from pileup, as derived for AK4 jets clustered after removing all charged particles assigned to a pileup vertex. Jet energies in simulated events are smeared to match the jet energy resolution (JER) observed in data. The XCone subjets are corrected with the same procedure as in Ref. [36], where an additional XCone correction is derived because of residual differences to AK4 jets. The correction is obtained from simulated samples of $\hbox {t}\overline{\hbox {t}}$ in the all-jets channel and parametrised as a function of the XCone subjet $p_{\textrm{T}}$ and $|\eta |$. The XCone jet mass is calibrated as described in Sect. 7. The JMS correction is applied to the four-momentum of the jet such that it changes only the mass but leaves the three-momentum unaltered.

## Particle-level phase space

The measurement of $m_{\textrm{jet}}$ is carried out at the particle level in the fiducial region defined below. The particle level is defined by the set of all stable particles, i.e. with a lifetime longer than $10^{-8}\,\text {s}$ as provided by the event simulation. We develop an unfolding procedure to correct the data for detector and pileup effects. This procedure provides a measurement at the particle level.

**Fig. 1:**
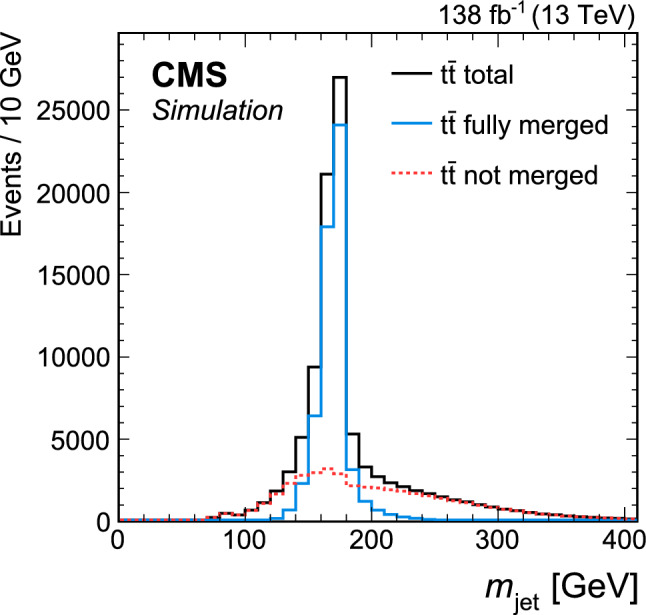
Distribution in $m_{\textrm{jet}}$ at the particle level after the selection of the fiducial region in the lepton+jets channel of $\hbox {t}\overline{\hbox {t}}$, simulated with powheg. The contributions from fully merged events (blue solid) and not merged events (red dashed) are displayed, as well as the sum of the two (black solid)

The fiducial region at the particle level is defined such that similar requirements can be used on data at the detector level, which helps to keep the corrections small in the unfolding step. In order to select the lepton+jets channel of the $\hbox {t}\overline{\hbox {t}}$ process, exactly one prompt electron or muon with $p_{\textrm{T}} >60\,\text {Ge}\hspace{-.08em}\text {V}$ originating from the decay of a W boson must be present. Decays to ${\uptau }$ leptons contribute a small background. They are not selected and are treated as background in this analysis. The two-step XCone clustering procedure is performed similarly to the one at the reconstruction level, as explained in Sect. 4, with all stable particles except for neutrinos as input. Decays of boosted top quarks must have an XCone jet with $p_{\textrm{T}} >400\,\text {Ge}\hspace{-.08em}\text {V}$. All three XCone subjets have to satisfy $p_{\textrm{T}} >30\,\text {Ge}\hspace{-.08em}\text {V}$ and $|\eta |<2.5$. The requirement on $|\eta |$ ensures that the XCone jet is reconstructed within the geometric acceptance of the detector. The second XCone jet has to have $p_{\textrm{T}} >10\,\text {Ge}\hspace{-.08em}\text {V}$ after the lepton four-momentum has been subtracted. This requirement rejects pathological cases, where the second XCone jet does not contain the b subjet from the $\textrm{t}$ decay. We find that 6.7% of all events are rejected by this requirement. The XCone-jet mass $m_{\textrm{jet}}$ has to be larger than the invariant mass of the sum of the second XCone jet and the selected lepton. Since the neutrino from the leptonic decay is not reconstructed, this requirement is always fulfilled if all decay products of the hadronic decay are reconstructed within the XCone jet, referred to as “fully merged events”. This criterion helps to select fully merged decays without introducing a bias on the measurement XCone jet, which would be the case with additional requirements on its substructure. It removes about 32.6% of the $\hbox {t}\overline{\hbox {t}}$ events at the particle level, where a large fraction of the removed events consists of not fully merged events. Figure 1 shows the distribution in $m_{\textrm{jet}}$ at the particle level after the selection of the fiducial region. The distribution has a narrow peak, with the maximum close to $m_{\textrm{t}}$. Contributions from the UE and FSR lead to a shift of the peak towards higher values. In the peak region, the contribution of fully merged top quark decays is about 87%. Contributions from $\hbox {t}\overline{\hbox {t}}$ events that are not fully merged dominate the regions to the left and right of the peak. Typically, in these events the top quark has only been partially reconstructed within the XCone jet, or the XCone jet originates from radiation not associated with the $\hbox {t}\overline{\hbox {t}}$ system. With respect to the measurement at 8$\,\text {Te}\hspace{-.08em}\text {V}$ [35], which used Cambridge–Aachen jets [83, 84] with $R=1.2$ and no grooming, the width of the distribution in the peak region is reduced by a factor of two. This improvement is achieved by the two-step XCone clustering procedure which acts as a grooming algorithm [33], removing all particles in the XCone jet not clustered into the three subjets.

## Event selection

At the detector level, the event selection aims to include a similar phase space as selected at the particle level. Events must contain a single muon or single electron with $p_{\textrm{T}} >60\,\text {Ge}\hspace{-.08em}\text {V}$ and $|\eta |<2.4$. Leptons with $55<p_{\textrm{T}} <60\,\text {Ge}\hspace{-.08em}\text {V}$ and $|\eta |<2.4$ are used to construct a sideband region when unfolding the data, as described in Sect. 9. Electrons with $p_{\textrm{T}} <120\,\text {Ge}\hspace{-.08em}\text {V}$ must pass an isolation requirement [79], where the isolation is defined as the $p_{\textrm{T}}$ sum of charged hadrons and neutral particles in a cone with radius $R=0.3$ around the electron. The isolation variable is corrected to mitigate the contribution from pileup. Electron candidates with $p_{\textrm{T}} >120\,\text {Ge}\hspace{-.08em}\text {V}$ and muons are rejected if there is an AK4 jet within ${\varDelta }R<0.4$ and $p_{\textrm{T}}^{\textrm{rel}} <40\,\text {Ge}\hspace{-.08em}\text {V}$, where $p_{\textrm{T}}^{\textrm{rel}}$ is the component of the lepton momentum orthogonal to the AK4-jet axis. The last criterion has high efficiency of selecting highly boosted $\textrm{t} \rightarrow {\text {b}} {\text {W}} (\rightarrow {\ell } {{\upnu }} _{\!\ell })$ decays, where the lepton would not have passed an isolation requirement because of the angular proximity of the b jet, while rejecting QCD multijet events [85, 86].

In order to suppress non-$\hbox {t}\overline{\hbox {t}}$ backgrounds, at least one AK4 jet is required to be b tagged using the DeepJet algorithm [87, 88]. The candidate b jets are required to have $p_{\textrm{T}} >30\,\text {Ge}\hspace{-.08em}\text {V}$ and $|\eta |<2.4$, and must pass a selection on the DeepJet discriminator value corresponding to a misidentification rate of 0.1% for light-flavour quark and gluon jets, and an efficiency of 68%.

**Fig. 2:**
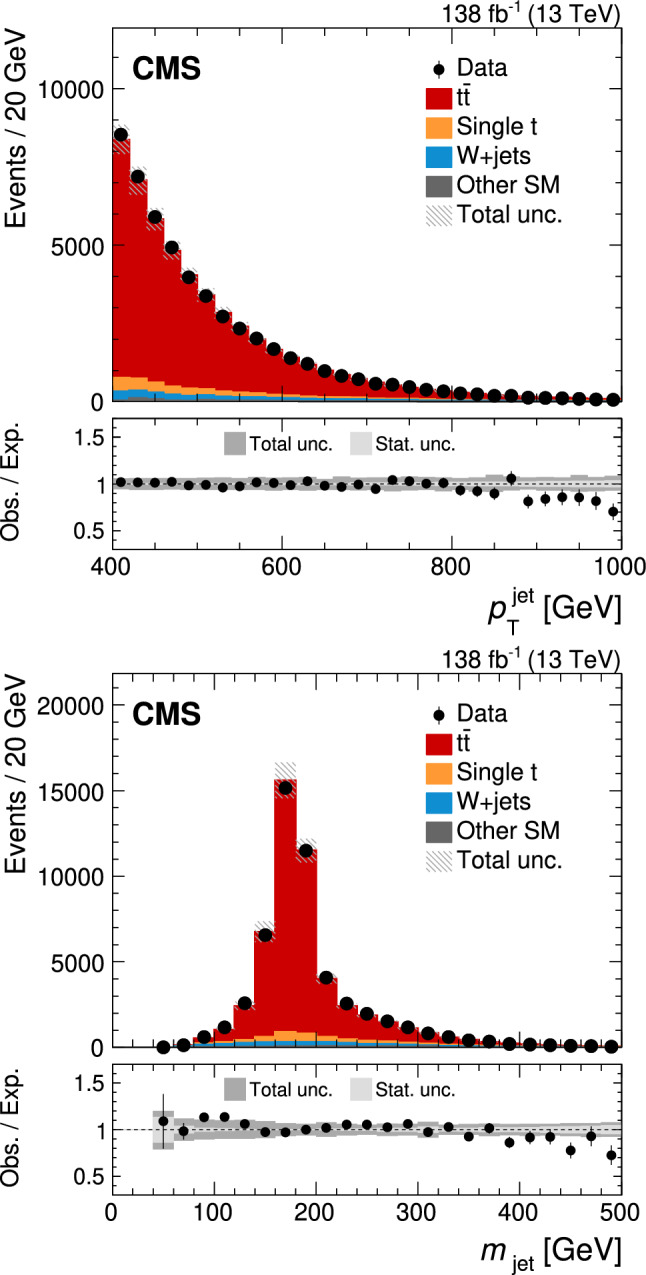
Distributions in the reconstructed XCone jet $p_{\textrm{T}}$  (upper) and $m_{\textrm{jet}}$  (lower), after the full event selection. The vertical bars on the markers show the statistical uncertainty. The hatched regions show the total uncertainty in the simulation, including the statistical and experimental systematic uncertainties. The lower panels show the ratio of the data to the simulation. The uncertainty bands include the experimental systematic uncertainties and statistical uncertainties in the simulation. In the ratios, the statistical (light grey) and total (dark grey) uncertainties are shown separately

In addition, the magnitude of the negative vector sum of the transverse momenta of the PF candidates in an event [89], $p_{\textrm{T}} ^{\textrm{miss}}$, has to be larger than 50$\,\text {Ge}\hspace{-.08em}\text {V}$. The energy scale corrections applied to AK4 jets are propagated to $p_{\textrm{T}} ^{\textrm{miss}}$. This requirement suppresses the contribution of multijet backgrounds from the production of light-flavour quarks and gluons.

The XCone jet is required to have $p_{\textrm{T}} >400\,\text {Ge}\hspace{-.08em}\text {V}$ and all three subjets must have $p_{\textrm{T}} >30\,\text {Ge}\hspace{-.08em}\text {V}$ and $|\eta |<2.5$. The second XCone jet has to have $p_{\textrm{T}} >10\,\text {Ge}\hspace{-.08em}\text {V}$ and the invariant mass of the system containing the second XCone jet and the lepton must not surpass $m_{\textrm{jet}}$.

**Fig. 3:**
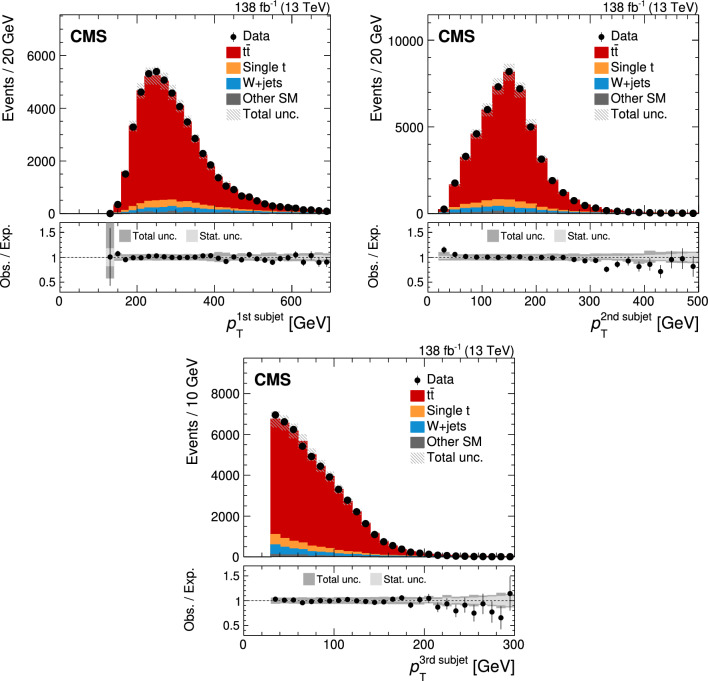
Distributions in reconstructed $p_{\textrm{T}}$ of the $p_{\textrm{T}}$-leading XCone subjet (upper left), second XCone subjet (upper right) and third XCone subjet (lower). The vertical bars on the markers show the statistical uncertainty. The hatched regions show the total uncertainty in the simulation, including the statistical and experimental systematic uncertainties. The lower panels show the ratio of the data to the simulation. The uncertainty bands include the experimental systematic uncertainties and statistical uncertainties in the simulation. In the ratios, the statistical (light grey) and total (dark grey) uncertainties are shown separately

Figure 2 shows the XCone jet $p_{\textrm{T}}$  (upper) and $m_{\textrm{jet}}$  (lower) spectra at the detector level. Here, data from all three years and both lepton flavours are combined. For the sake of comparing the shapes of these distributions, the $\hbox {t}\overline{\hbox {t}}$ simulation has been scaled down such that the number of simulated events matches the number of events observed in the data. The distribution in $p_{\textrm{T}}$ shows the characteristic falling behaviour above the 400$\,\text {Ge}\hspace{-.08em}\text {V}$ threshold, while the distribution in $m_{\textrm{jet}}$ shows a narrow peak close to $m_{\textrm{t}}$. We find reasonable agreement between data and simulation in the $p_{\textrm{T}}$ and $m_{\textrm{jet}}$ distributions when we use the JECs, and the XCone and JMS corrections described in Sect. 7. For $p_{\textrm{T}}$ above 900$\,\text {Ge}\hspace{-.08em}\text {V}$, we observe that the simulation predicts more events than observed in data, a feature which has been reported previously in differential $\hbox {t}\overline{\hbox {t}}$ cross section measurements when comparing to NLO calculations [90–92]. Figure 3 shows the distributions in $p_{\textrm{T}}$ of the XCone subjets. Because of the XCone-jet selection with $p_{\textrm{T}} >400\,\text {Ge}\hspace{-.08em}\text {V}$, the first subjet has a most probable $p_{\textrm{T}}$ of about 250$\,\text {Ge}\hspace{-.08em}\text {V}$, and the second subjet has a value of about 150$\,\text {Ge}\hspace{-.08em}\text {V}$. The remaining subjet features a falling distribution, starting from the minimum value of 30$\,\text {Ge}\hspace{-.08em}\text {V}$.

## Calibration of the jet mass scale

The experimental precision in the measurement of $m_{\textrm{jet}}$ in boosted top quark decays is limited by the calibration of the jet four-momentum. In our previous analysis [36], the uncertainty in the JES was propagated to $m_{\textrm{jet}}$ and resulted in the dominant experimental systematic uncertainty. In this article, we measure the JMS using the invariant mass of the two XCone subjets originating from the hadronic W boson decay. With this additional measurement, the uncertainty in the JES affects the jet three-momentum, while the uncertainty in the JMS affects $m_{\textrm{jet}}$. The JMS calibration is crucial for the improvement in the overall precision of this measurement.

**Fig. 4:**
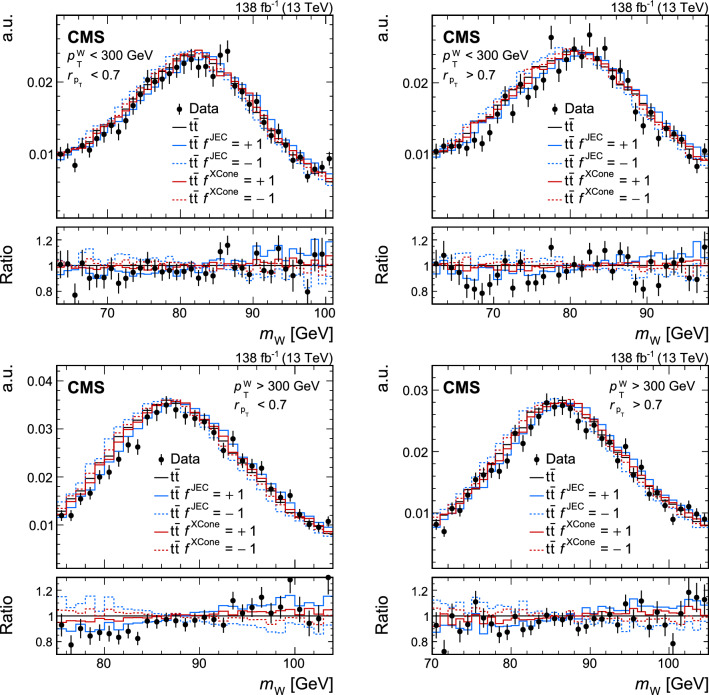
Peak region of the reconstructed W boson mass in the four regions $p_{\textrm{T}} ^{{\text {W}}} <300\,\text {Ge}\hspace{-.08em}\text {V}$ and $r_{p_{\textrm{T}}} <0.7$ (upper left), $p_{\textrm{T}} ^{{\text {W}}} <300\,\text {Ge}\hspace{-.08em}\text {V}$ and $r_{p_{\textrm{T}}} >0.7$ (upper right), $p_{\textrm{T}} ^{{\text {W}}} >300\,\text {Ge}\hspace{-.08em}\text {V}$ and $r_{p_{\textrm{T}}} <0.7$ (lower left), and $p_{\textrm{T}} ^{{\text {W}}} >300\,\text {Ge}\hspace{-.08em}\text {V}$ and $r_{p_{\textrm{T}}} >0.7$ (lower right). The background-subtracted data and the $\hbox {t}\overline{\hbox {t}}$ simulation are normalised to unit area. For illustration, the $\hbox {t}\overline{\hbox {t}}$ simulation is also shown with the JEC and XCone correction factors varied by one standard deviation. The lower panels show the ratios to the nominal $\hbox {t}\overline{\hbox {t}}$ simulation

For the JMS calibration, the same selection as for the measurement is applied. The W boson decay is reconstructed using two of the three XCone subjets from the XCone jet initiated by the hadronic top quark decay. We identify the XCone subjet originating from the fragmentation of the b quark using the DeepJet algorithm on AK4 jets. First, the AK4 jet with the largest value of the DeepJet b discriminant among those with angular distance ${\varDelta }R<1.2$ to the XCone jet is selected. In a second step, the XCone subjet with the smallest ${\varDelta }R$ to the selected b-tagged AK4 jet is assigned to originate from the b quark. This XCone subjet is rejected, and the measurement of the JMS is performed using the invariant mass of the other two XCone subjets. Data from the two lepton flavours and three different years are combined for the JMS calibration.

The JMS in simulation is adjusted by introducing two factors, $f^{\textrm{JEC}}$ and $f^{\textrm{XCone}}$, that vary the jet energy scale in the AK4 JECs and the additional XCone-jet corrections, respectively. The factors are constructed such that values of 0, ${+}1$ and ${-}1$ represent the nominal correction, and the up and down shifts by one standard deviation, respectively. With these two factors, the squared XCone jet mass becomes

1 $$\begin{aligned} m_{\textrm{jet}} ^2= & {} \left( \sum _{i=1}^3 p_i \left( c_{\textrm{JEC}} (p_{{\textrm{T}},i}, \eta _i) + f^{\textrm{JEC}} \sigma _{\textrm{JEC}} (p_{{\textrm{T}},i}, \eta _i) \right) \right. \nonumber \\{} & {} \times \left. \left( c_{\textrm{XC}} (p_{{\textrm{T}},i}, \eta _i) + f^{\textrm{XCone}} \sigma _{\textrm{XC}} (p_{{\textrm{T}},i}, \eta _i) \right) \right) ^2, \end{aligned}$$

where $p_i$ are the three subjet four-momenta before the application of the JEC and XCone corrections, $c_{\textrm{JEC}} (p_{{\textrm{T}},i}, \eta _i)$ and $c_{\textrm{XC}} (p_{{\textrm{T}},i}, \eta _i)$ denote the JEC and XCone corrections, respectively, and $\sigma _{\textrm{JEC}} (p_{{\textrm{T}},i}, \eta _i)$ and $\sigma _{\textrm{XC}} (p_{{\textrm{T}},i}, \eta _i)$ are the uncertainties in these corrections. The JES and XCone corrections and the corresponding uncertainties depend on the $p_{\textrm{T}}$ and $\eta$ of the uncorrected subjet four-momentum. The additional corrections proportional to $f^{\textrm{JEC}}$ and $f^{\textrm{XCone}}$ allow $m_{\textrm{jet}}$ to float, while retaining the shape and functional form of the JEC and XCone uncertainties in $p_{\textrm{T}}$ and $\eta$. This JMS correction is constructed to change only the XCone jet mass but not the three-momentum that is calibrated with established methods. The decoupling of the JMS correction from the three-momentum calibration allows the JMS correction to target effects which change only the jet mass and not the three-momentum, like splitting and merging of calorimeter clusters.

The measurement is performed in four regions that are defined in the two-dimensional plane of the $p_{\textrm{T}}$ of the reconstructed W boson, $p_{\textrm{T}} ^{{\text {W}}}$, and the ratio $r_{p_{\textrm{T}}} = p_{\textrm{T}} ^{\text {s}_1}/p_{\textrm{T}} ^{{\text {W}}}$, defined as the ratio of the $p_{\textrm{T}}$ carried by the highest $p_{\textrm{T}}$ XCone subjet $\text {s}_1$ to $p_{\textrm{T}} ^{{\text {W}}}$. These regions are constructed to reduce correlations between $f^{\textrm{JEC}}$ and $f^{\textrm{XCone}}$, because these factors can cancel each other in an inclusive measurement of the JMS. We find an improvement by a factor of 1.6 in the obtained precision of $f^{\textrm{JEC}}$ and $f^{\textrm{XCone}}$ when using these four regions, compared to a calibration using the inclusive $m_{{\text {W}}}$ distribution. Because of the different size of $\sigma _{\textrm{JEC}} (p_{\textrm{T}}, \eta )$ and $\sigma _{\textrm{XC}} (p_{\textrm{T}}, \eta )$ in subjet $p_{\textrm{T}}$ and $\eta$, the effects of $f^{\textrm{JEC}}$ and $f^{\textrm{XCone}}$ are different in the four regions defined by $p_{\textrm{T}} ^{{\text {W}}}$ and $r_{p_{\textrm{T}}}$, such that these two factors can be determined simultaneously. Figure 4 shows the four distributions in the reconstructed W boson mass $m_{{\text {W}}}$ in the vicinity of their peaks in the four regions, defined by $p_{\textrm{T}} ^{{\text {W}}}$ larger or smaller than 300$\,\text {Ge}\hspace{-.08em}\text {V}$ and $r_{p_{\textrm{T}}}$ larger or smaller than 0.7. We consider only regions around the peak position with bins populated by more than 100 events in the background-subtracted data for a bin width of 1$\,\text {Ge}\hspace{-.08em}\text {V}$. This requirement leads to the following $m_{{\text {W}}}$ ranges in the four regions: 70–105$\,\text {Ge}\hspace{-.08em}\text {V}$ for $p_{\textrm{T}} ^{{\text {W}}} >300\,\text {Ge}\hspace{-.08em}\text {V}$ and $r_{p_{\textrm{T}}} >0.7$; 75–104$\,\text {Ge}\hspace{-.08em}\text {V}$ for $p_{\textrm{T}} ^{{\text {W}}} >300\,\text {Ge}\hspace{-.08em}\text {V}$ and $r_{p_{\textrm{T}}} <0.7$; 62–98$\,\text {Ge}\hspace{-.08em}\text {V}$ for $p_{\textrm{T}} ^{{\text {W}}} <300\,\text {Ge}\hspace{-.08em}\text {V}$ and $r_{p_{\textrm{T}}} >0.7$; and 63–101$\,\text {Ge}\hspace{-.08em}\text {V}$ for $p_{\textrm{T}} ^{{\text {W}}} <300\,\text {Ge}\hspace{-.08em}\text {V}$ and $r_{p_{\textrm{T}}} <0.7$. These ranges are used to exclude tails in the $m_{{\text {W}}}$ distributions, which originate from a wrong assignment of subjets to the reconstructed W boson. In total, 138 bins are used in the JMS calibration. The distributions of background-subtracted data and $\hbox {t}\overline{\hbox {t}}$ signal have been normalised to unit area and are given in arbitrary units (a.u.), such that only shapes are considered and the total yield does not affect the measurement. The $\hbox {t}\overline{\hbox {t}}$ simulation is shown for the different variations in the jet corrections, parametrised by $f^{\textrm{JEC}}$ and $f^{\textrm{XCone}}$. The peak in $m_{{\text {W}}}$ is shifted in the four regions by 0.42—0.61$\,\text {Ge}\hspace{-.08em}\text {V}$ and by 0.17—0.25$\,\text {Ge}\hspace{-.08em}\text {V}$ for the $f^{\textrm{JEC}}$ and $f^{\textrm{XCone}}$ variations, respectively.

In each bin *i* of the $m_{{\text {W}}}$ distribution, a linear prediction $g_i$ as a function of $f^{\textrm{JEC}}$ and $f^{\textrm{XCone}}$ is defined,

2 $$\begin{aligned} g_i(f^{\textrm{JEC}},f^{\textrm{XCone}}) = a_i + b_i f^{\textrm{JEC}} + c_i f^{\textrm{XCone}}, \end{aligned}$$

with the free parameters $a_i$, $b_i$, and $c_i$. The free parameters are obtained from a fit to simulation in the $f^{\textrm{JEC}}$-$f^{\textrm{XCone}}$ plane in each bin *i*. We have verified that a linear fit in both factors describes the dependence of $m_{{\text {W}}}$ on $f^{\textrm{JEC}}$ and $f^{\textrm{XCone}}$ sufficiently well, with a fit quality matching the expectation of statistical fluctuations only.

To verify that the statistical uncertainties in the simulation do not bias the result, we have performed a test where we increased the bin size in the four $m_{{\text {W}}}$ distributions by a factor of three, to 3$\,\text {Ge}\hspace{-.08em}\text {V}$. This results in 47 bins and reduces the fluctuations in the four $m_{{\text {W}}}$ distributions. We find that the linear parametrisations of Eq. (2) provide a good description of the variations in $f^{\textrm{JEC}}$ and $f^{\textrm{XCone}}$. Performing the JMS calibration with these larger bins and reduced statistical uncertainties in $g_i$ gives a similar result with respect to the nominal fit with 138 bins. The reduced information in the fit with 47 bins results in an increased correlation between $f^{\textrm{XCone}}$ and $f^{\textrm{JEC}}$ compared to the nominal fit.

**Fig. 5:**
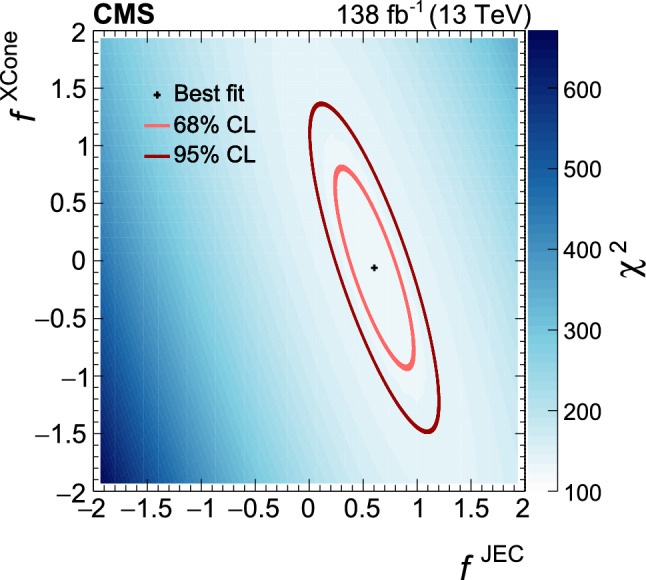
The two-dimensional $\chi ^2$ as a function of $f^{\textrm{JEC}}$ and $f^{\textrm{XCone}}$, obtained from a comparison of background-subtracted data with the predictions from $\hbox {t}\overline{\hbox {t}}$ production in the reconstructed $m_{{\text {W}}}$ distributions. The minimum is indicated by a black cross, and the borders of the 68 and 95% $\text {CL}$ intervals are shown by the light and dark red ellipses, respectively

The factors $f^{\textrm{JEC}}$ and $f^{\textrm{XCone}}$ are obtained from a fit to the data, where a two-dimensional $\chi ^2$ function is constructed,

3 $$\begin{aligned} \chi ^2 = d^T V^{-1} d. \end{aligned}$$

The vector *d* is built from the differences between the predictions $g_i(f^{\textrm{JEC}},f^{\textrm{XCone}})$ and the background-subtracted data in each bin *i* of all four regions in $p_{\textrm{T}} ^{{\text {W}}}$ and $r_{p_{\textrm{T}}}$. The covariance matrix *V* includes the statistical uncertainty in data, also considering correlations from the normalisation to unit area, and the uncertainties in the functions $g_i$ from the fit to simulation. The latter are estimated from the statistical uncertainty of the simulated $\hbox {t}\overline{\hbox {t}}$ sample. We also include the leading systematic uncertainties, namely the JER uncertainty, modelling uncertainties from the $\hbox {t}\overline{\hbox {t}}$ simulation, and uncertainties from the background subtraction. These uncertainties are treated as fully correlated across all bins as well as the four regions. We find that the statistical uncertainties are the dominant uncertainties in the calibration of the JMS, followed by the JER uncertainties. All other uncertainties are small in comparison.

Figure 5 shows the evaluated two-dimensional $\chi ^2$, as a function of $f^{\textrm{JEC}}$ and $f^{\textrm{XCone}}$. The minimum of the $\chi ^2$ function lies within the one-standard deviation intervals of the correction factors. The global minimum has a value of $\chi ^2 = 130$ for 132 degrees of freedom. We find the best-fit values $f^{\textrm{JEC}} = 0.60 \pm 0.24$ and $f^{\textrm{XCone}} = -0.06 \pm 0.57$ with a linear correlation coefficient of ${-}0.66$. The JMS uncertainty obtained from the two-dimensional 68% confidence level ($\text {CL}$) interval is reduced compared to the variations of $f^{\textrm{JEC}}$ and $f^{\textrm{XCone}}$ in the intervals between ${-}1$ and ${+}1$. In order to construct variations of one standard deviation in one dimension for the evaluation of systematic uncertainties, the endpoints of the minor axis are chosen. These result in the largest shift in the $m_{\textrm{jet}}$ distribution, because along the minor axis both factors $f^{\textrm{JEC}}$ and $f^{\textrm{XCone}}$ shift the value of $m_{\textrm{jet}}$ in the same direction. Changes of $f^{\textrm{JEC}}$ and $f^{\textrm{XCone}}$ along the major axis result in shifts in opposite directions, which cancel to a large part. The extracted value pairs in $(f^{\textrm{JEC}}, f^{\textrm{XCone}})$, with the nominal value pair of $(0.60, -0.06)$, are (0.78, 0.01) and $(0.42, -0.13)$, which are used in the determination of systematic uncertainties. These pairs of values are referred to as JMS correction in the following, with the corresponding uncertainties. We have verified that variations of $m_{\textrm{t}}$ in the $\hbox {t}\overline{\hbox {t}}$ simulation do not alter this result. Additionally, we have tested that the results obtained from the electron and muon channels are compatible. The final results of the $m_{\textrm{jet}}$ measurement agree within the uncertainties if the JMS calibration is carried out in the electron channel and applied to the muon channel, and vice versa.

**Fig. 6:**
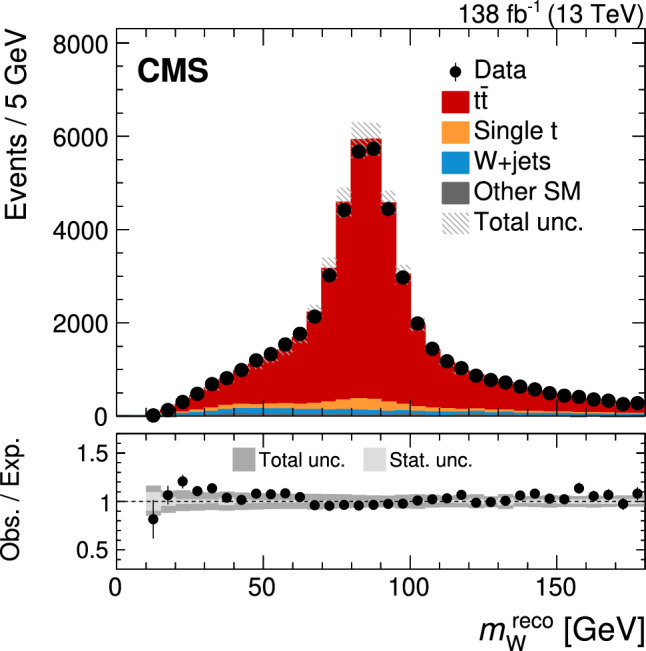
Jet mass distribution of hadronic decays of the W boson, reconstructed from two XCone subjets. The vertical bars on the markers show the statistical uncertainty. The hatched regions show the total uncertainty in the simulation, including the statistical and experimental systematic uncertainties. The lower panel shows the ratio of the data to the simulation. The uncertainty bands include the experimental systematic uncertainties and statistical uncertainties in the simulation. The statistical (light grey) and total (dark grey) uncertainties are shown separately in the ratio

**Fig. 7:**
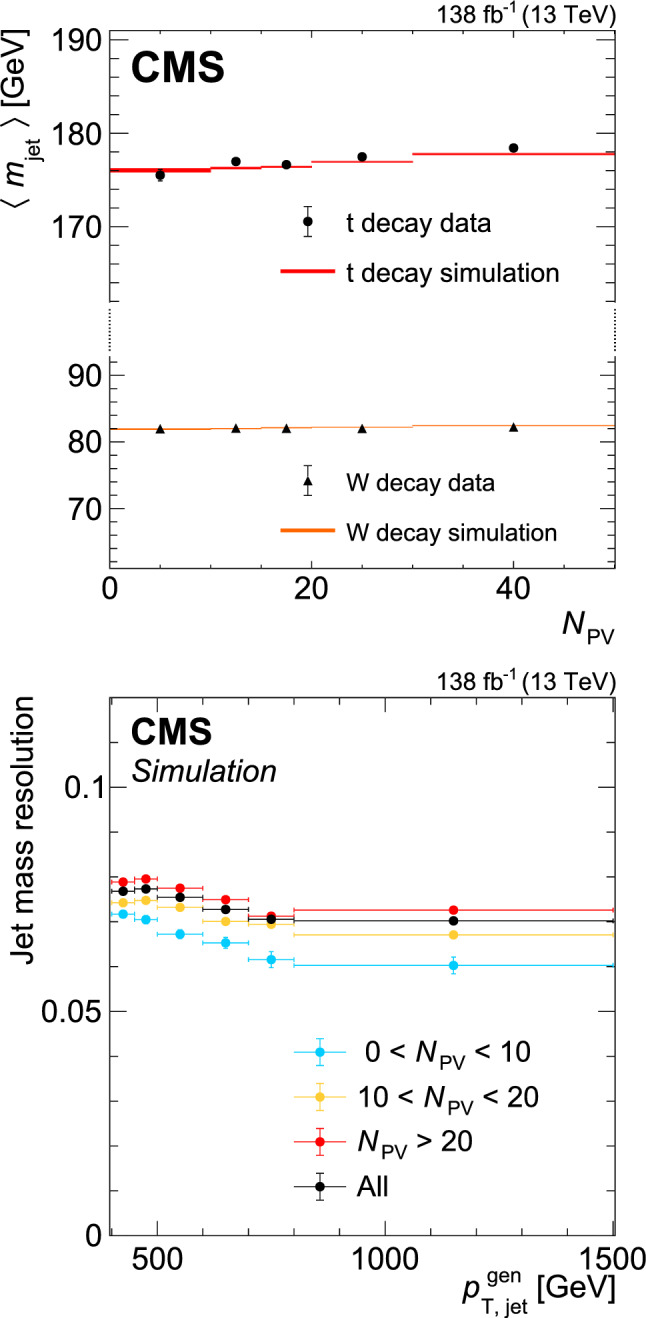
Mean values of the $m_{\textrm{jet}}$ distribution for $\textrm{t}$ and W boson decays, as a function of the number of primary vertices $N_{\text {PV}}$ (upper). Data (markers) are compared with $\hbox {t}\overline{\hbox {t}}$ simulation (filled areas). The vertical bars and size of the filled areas show the statistical uncertainties in the calculation of the mean values. Jet mass resolution in simulation as a function of particle-level XCone-jet $p_{\textrm{T}}$, given for different intervals in the number of primary vertices (lower). The vertical bars indicate the statistical uncertainties and the horizontal bars indicate the bin width

Figure 6 shows the reconstructed $m_{{\text {W}}}$ distribution after applying the JMS correction. The data are well described by the simulation over the full distribution in $m_{\textrm{jet}}$. The mean values of $m_{\textrm{jet}}$ for the reconstructed top quark and W boson masses are shown as a function of the number of primary vertices in Fig. 7 (upper). The values for the top quark mass are obtained using all three XCone subjets, while the W boson mass is calculated from the two subjets not matched to the b-tagged AK4 jet. The mean values of $m_{\textrm{jet}}$ are larger than the parameters $m_{\textrm{t}}$ and $m_{{\text {W}}}$ used in the simulation by about 4 and 2$\,\text {Ge}\hspace{-.08em}\text {V}$, respectively, because of contributions from the UE and pileup interactions. The slope of the mean value of $m_{\textrm{jet}}$ as a function of the number of pileup interactions is small, indicating that the XCone reconstruction and calibration remove most of the contributions from pileup. The mean values and the slopes are well described by the simulation. The achieved resolution in $m_{\textrm{jet}}$ is displayed in Fig. 7 (lower). We calculate the resolution as the width parameter of a Gaussian function, fitted to distributions in $m_{\textrm{jet}} ^\text {rec} / m_{\textrm{jet}} ^\text {gen}$, where $m_{\textrm{jet}} ^\text {rec}$ denotes the reconstructed value of $m_{\textrm{jet}}$ at the detector level and $m_{\textrm{jet}} ^\text {gen}$ is the jet mass at the particle level. The achieved resolution is below 8% over the full range in $p_{\textrm{T}}$. For an inclusive selection in the number of primary vertices, the mass resolution improves from 7.7% at $p_{\textrm{T}} =400\,\text {Ge}\hspace{-.08em}\text {V}$ to 7% for $p_{\textrm{T}} >800\,\text {Ge}\hspace{-.08em}\text {V}$. For a selection with less than 10 primary vertices, the resolution is about one percentage point better than for a selection with more than 20 primary vertices.

## Studies of the final state radiation

The uncertainty in the modelling of FSR was the dominant model uncertainty in the previous $m_{\textrm{jet}}$ measurement at 13$\,\text {Te}\hspace{-.08em}\text {V}$ [36]. There, the energy scale parameter $\mu$, which enters into the definition of the strong coupling $\alpha _{\textrm{S}} ^{{\textrm{FSR}}}(\mu ^{2})$, was changed by factors of 0.5 and 2 in the FSR simulation to estimate this uncertainty. This is equivalent to changing the value of the effective strong coupling at the mass of the Z boson from $\alpha _{\textrm{S}} ^{{\textrm{FSR}}}(m_{{\hbox {Z}}}^{2}) =0.1365$, as used in the parton shower and UE event tune CUETP8M2T4 for the simulation of 2016 data, to values of 0.1556 and 0.1217, respectively. While the data are well described using the central value, we find that the large uncertainty variations do not describe the data in the fiducial region of this measurement. For the simulation of 2017 and 2018 data, the CP5 tune is used with $\alpha _{\textrm{S}} ^{{\textrm{FSR}}}(m_{{\hbox {Z}}}^{2}) =0.118$, which is not the optimal choice for the modelling of jet substructure observables in $\hbox {t}\overline{\hbox {t}}$ production, where a larger value is preferred [69]. To remedy this situation, we perform a study of the FSR modelling and find the value of $\alpha _{\textrm{S}} ^{{\textrm{FSR}}}(m_{{\hbox {Z}}}^{2})$ that fits the data best. The study is performed separately for the two samples with different tunes, namely for the year 2016, and for the combination of the years 2017 and 2018. The uncertainties in $\alpha _{\textrm{S}} ^{{\textrm{FSR}}}(m_{{\hbox {Z}}}^{2})$ from this study are propagated to the FSR uncertainty in the $m_{\textrm{jet}}$ measurement.

As a starting point, we modify the energy scale in the FSR simulation by a factor $f_{\textrm{FSR}}$. With this definition, the FSR modelling uncertainty as used in the previous measurement is obtained by setting $f_{\textrm{FSR}} =0.5$ and 2. The prediction becomes a function of $f_{\textrm{FSR}}$, which we use to determine the best fit value of $f_{\textrm{FSR}}$ through a comparison of distributions between data and simulation in the *N*-subjettiness ratio $\tau _{32} =\tau _3 / \tau _2$ [39, 40]. The distributions in $\tau _{32}$ are sensitive to the angular distribution of the energy density inside jets and are thus well suited for determining $f_{\textrm{FSR}}$.

We use the same event selection as used for the $m_{\textrm{jet}}$ measurement, described in Sect. 6. Instead of XCone jets, we use AK8 jets to study the $\tau _{32}$ distributions. These have a higher sensitivity to effects from FSR, because AK8 jets are obtained without jet grooming, unlike the XCone jets clustered with the two-step procedure. The AK8 jet that is within ${\varDelta }R < 0.8$ of the XCone jet is selected, provided it has $m_{\textrm{jet}} >140\,\text {Ge}\hspace{-.08em}\text {V}$. This requirement on $m_{\textrm{jet}}$ ensures that only jets including all particles from the hadronic $\textrm{t}$ decay are accepted.

**Fig. 8:**
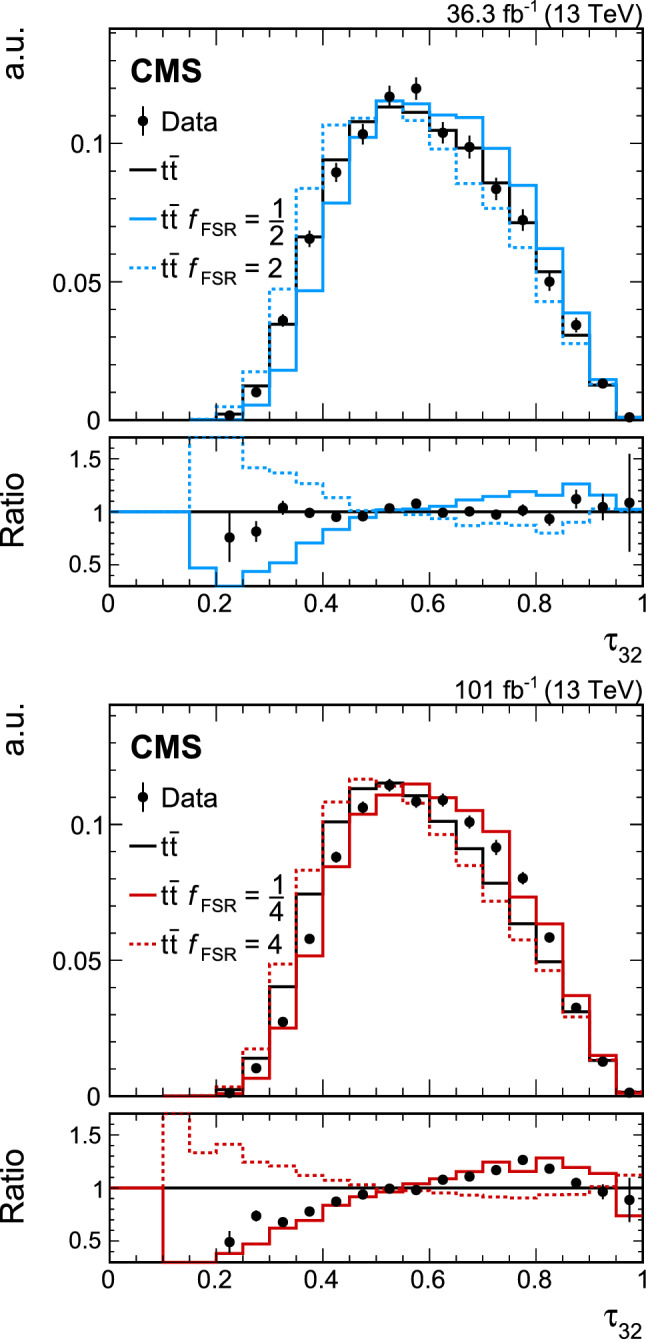
The normalised distributions in $\tau _{32}$ for AK8 jets with $m_{\textrm{jet}} >140\,\text {Ge}\hspace{-.08em}\text {V}$ from the hadronic decay of boosted top quarks. Shown are distributions for 2016 (upper) and the combination of 2017 and 2018 (lower). The background-subtracted data are compared to $\hbox {t}\overline{\hbox {t}}$ simulations with the UE tunes CUETP8M2T4 for 2016 and CP5 for the combination of 2017 and 2018, and different values of $f_{\textrm{FSR}}$ are shown as well. The lower panels show the ratio to the $\hbox {t}\overline{\hbox {t}}$ simulation with $f_{\textrm{FSR}} =1$

Figure 8 shows the normalised distributions in $\tau _{32}$ for 2016 (upper), and the combination of 2017 and 2018 (lower). In both cases, larger values of $f_{\textrm{FSR}}$ shift the distributions to lower values in $\tau _{32}$, and smaller values of $f_{\textrm{FSR}}$ lead to a larger average value of $\tau _{32}$. This is compatible with the expectation of less radiation for larger values of $f_{\textrm{FSR}}$, corresponding to smaller values of $\alpha _{\textrm{S}} ^{\textrm{FSR}}$. Without additional radiation, $\tau _{32}$ becomes small and compatible with a three-prong decay. If radiation is added to the jet, the value of $\tau _3$ increases, and shifts the average $\tau _{32}$ to larger values.

The sensitivity of the $\tau _{32}$ distribution to FSR can be used to determine the value of $f_{\textrm{FSR}}$ that is most compatible with the data. We construct predictions $g_i(f_{\textrm{FSR}})$ in each bin *i* of the normalised $\tau _{32}$ distributions,

4 $$\begin{aligned} g_i(f_{\textrm{FSR}}) = a_i + b_i \log {f_{\textrm{FSR}} ^{-2}} + c_i f_{\textrm{FSR}} ^{-2}, \end{aligned}$$

with the free parameters $a_i$, $b_i$, and $c_i$. The functional form of $g_i$ is inspired by the logarithmic dependence of $\alpha _{\textrm{S}} ^{\textrm{FSR}}$ on the square of the modified energy scale $(f_{\textrm{FSR}} \mu )^2$. The values of the free parameters are determined in a fit to simulation, sampled at the points $f_{\textrm{FSR}} \in \{\frac{1}{2}, 1, 2\}$ in 2016 and $f_{\textrm{FSR}} \in \{ \frac{1}{4}, \frac{1}{2}, \frac{1}{\sqrt{2}}, 1, \sqrt{2}, 2, 4 \}$ in 2017 and 2018.

The compatibility with the data is tested with a $\chi ^2$ function, equivalent to the definition in Eq. (3). The vector of differences is built from the normalised background-subtracted data, and the predictions $g_i(f_{\textrm{FSR}})$. The uncertainties taken into account by the covariance matrix include statistical uncertainties from data with correlations from the normalisation, and systematic uncertainties in the JECs and in the predictions $g_i(f_{\textrm{FSR}})$. The latter are conservatively estimated by using the largest statistical uncertainty in a given bin *i* from any of the points obtained from the simulated samples with different values of $f_{\textrm{FSR}}$. This choice was made because the point with $f_{\textrm{FSR}} =4$ has the smallest statistical precision due to the presence of a large spread of weights in the simulation. The statistical uncertainty in data is the dominant uncertainty in this measurement.

The best fit value of $f_{\textrm{FSR}}$ is obtained by minimising the $\chi ^2$ function. Uncertainties corresponding to one standard deviation are evaluated at $\chi ^2_\text {min}+1$. We obtain the best fit values $f_{\textrm{FSR}} = 0.97 \pm 0.07$ for 2016, and $f_{\textrm{FSR}} = 0.33 \pm 0.02$ for the combined data of 2017 and 2018. The uncertainties in $f_{\textrm{FSR}}$ take into account statistical and leading systematic sources, where the latter are dominated by changes of the modelling in simulation, as described in Sect. 10. The modelling uncertainties included are uncertainties in the initial state radiation (ISR), the colour reconnection model, the underlying event tune, and the matching between matrix element and the parton shower. Experimental uncertainties considered are uncertainties in the JECs, the additional XCone-jet corrections, and JMS. We have found that the $\tau _{32}$ distributions obtained with different values of $m_{\textrm{t}}$ are compatible within the statistical precision of the simulated $\hbox {t}\overline{\hbox {t}}$ samples, and therefore we do not consider changes of $m_{\textrm{t}}$ in this study. We find that the statistical uncertainties from data and the limited size of the simulated samples constitute the largest source of uncertainty in the determination of $f_{\textrm{FSR}}$.

The best fit values of $f_{\textrm{FSR}}$ can be translated to values of $\alpha _{\textrm{S}}^{\textrm{FSR}} (m_\textrm{Z}^{2})$. This gives $\alpha _{\textrm{S}}^{\textrm{FSR}} (m_\textrm{Z}^{2}) = 0.1373_{-0.0018}^{+0.0017}$ for 2016 and $\alpha _{\textrm{S}}^{\textrm{FSR}} (m_\textrm{Z}^{2}) = 0.1416_{-0.0018}^{+0.0019}$ for the combination of 2017 and 2018, evaluated using five active flavours in the four-loop evolution of $\alpha _{\textrm{S}}$ [93]. We note that these values do not represent a generally valid measurement of $\alpha _{\textrm{S}} ^{\textrm{FSR}}$, which would need a different treatment of theory uncertainties from missing higher orders, but the results can be used to calibrate the two different tunes used for the $\hbox {t}\overline{\hbox {t}}$ simulation with powheg +pythia. In fact, the two values are compatible and much closer to each other than the values used in the CUETP8M2T4 and CP5 tunes. The uncertainty for 2016 is comparable to the one from the combination of 2017 and 2018, which constitutes a larger data set, because the latter is dominated by statistical uncertainties in the simulation originating from a large spread of weights used to obtain the samples with changes in $f_{\textrm{FSR}}$. The data are well described by the nominal simulation in 2016, but prefer a larger value of $\alpha _{\textrm{S}} ^{\textrm{FSR}}$ in the 2017 and 2018 simulations. We have checked that the 2017 and 2018 data are equally well or better described by the adjusted simulations with $f_{\textrm{FSR}} = 0.33$ in all distributions relevant for this analysis. The change in the 2016 simulation is insubstantial, with changes in distributions that are consistent with the statistical uncertainties of the simulated $\hbox {t}\overline{\hbox {t}}$ sample. We have verified that extracting $f_{\textrm{FSR}}$ from different intervals in $m_{\textrm{jet}}$ and $p_{\textrm{T}}$ leads to compatible results, validating the calibration of the FSR modelling in the full fiducial region of this measurement.

## Unfolding

The data are unfolded to the particle level using regularised unfolding as implemented in the TUnfold [94] framework. We have chosen the curvature regularisation condition, such that the second derivative of the unfolded result is regularised. This option introduces the smallest model dependencies in this measurement. The optimal regularisation strength is found by minimising the average global correlation coefficient in the output bins [95]. In addition to the measurement phase space defined in Sect. 6, five sideband regions are constructed by loosening the most important selection steps. These regions include events where the XCone jet has $350<p_{\textrm{T}} <400\,\text {Ge}\hspace{-.08em}\text {V}$, the lepton has $55<p_{\textrm{T}} <60\,\text {Ge}\hspace{-.08em}\text {V}$, at least one of the XCone subjets has $10<p_{\textrm{T}} <30\,\text {Ge}\hspace{-.08em}\text {V}$, $m_{\textrm{jet}}$ is less than the invariant mass of the sum of the second XCone jet and lepton, and the AK4 jet passes a b-tagging requirement with a misidentification rate of 1%, but not the tight requirement with 0.1%. Additionally, the measurement region and the region with XCone jet $350<p_{\textrm{T}} <400\,\text {Ge}\hspace{-.08em}\text {V}$ are divided into bins of $p_{\textrm{T}}$. The two bins in the peak region of $m_{\textrm{jet}}$ with bin boundaries at 152, 172 and 192$\,\text {Ge}\hspace{-.08em}\text {V}$ are split into four bins in the unfolding, but merged afterwards to avoid large bin-to-bin correlations. The splitting into regions of $p_{\textrm{T}} ^{\textrm{jet}}$ and the subdivision of $m_{\textrm{jet}}$ bins result in a reduced dependence on the modelling parameters in the $\hbox {t}\overline{\hbox {t}}$ simulation and help to reduce the corresponding uncertainties. In addition, this procedure ensures that the most important migrations between the detector and particle levels into and out of the fiducial region of the measurement are included in the unfolding and not purely estimated from simulation. In total, the response matrix includes 200 bins at the detector level and 72 bins at the particle level.

We unfold the three years individually in order to check for a potential bias originating from the different tunes in the $\hbox {t}\overline{\hbox {t}}$ simulation that is used to construct the response matrix. With the dedicated calibration of the FSR parameter in the simulation, all three years are compatible and agree within one standard deviation. We have also ensured that unfolding the electron and muon channels separately leads to a consistent result. For the final measurement, all data and simulated samples are combined before the unfolding.

## Uncertainties

Several sources of statistical and systematic uncertainties are considered in the measurement of $m_{\textrm{jet}}$. These are split into four categories: statistical, experimental, model, and theory uncertainties.

Statistical uncertainties are defined as the uncertainties due to the finite statistical precision of the data. With respect to the previous measurement [36], the statistical precision is increased by including data from 2017 and 2018, which increases the size of the data set by a factor of almost four. The statistical uncertainties are propagated through the unfolding process using Gaussian error propagation.

Experimental uncertainties encompass uncertainties in correction factors that are connected to the calibration of physics objects. These include the JECs [38], JER, additional XCone-jet corrections, JMS, as well as the factors correcting for the efficiencies in the trigger selection [47], lepton identification [78, 79], and b tagging [96]. The JMS correction has been obtained by calibrating $m_{\textrm{jet}}$ in the reconstructed $m_{{\text {W}}}$, which is dominated by XCone subjets originating from light-flavour quarks. To account for a possible difference in the detector response to XCone subjets originating from the fragmentation of b quarks, an additional flavour uncertainty [38] is applied to XCone subjets matched to AK4 b-tagged jets (JMS b flavour uncertainty), where the matching is identical to the procedure outlined in Sect. 7. This JMS b flavour uncertainty is obtained from the response difference of b jets in pythia and herwig [97, 98]. In addition, it is studied in a Z +b -jet sample where the b jet response can be studied in data [38]. The uncertainties in the reweighting of the pileup profile are considered. The experimental uncertainties are calculated by changing the corrections up and down by one standard deviation, and the difference with respect to the nominal response matrix is then propagated to the unfolded distribution. The uncertainty in the measurement of the integrated luminosity is estimated to be 1.6% [48–50] and is assigned to the unfolded distribution directly. Statistical uncertainties from the limited size of the simulated samples, denoted by “MC stat”, are included in the experimental uncertainties. The simulated samples for 2017 and 2018 increase the statistical precision of the unfolding compared to the previous measurement with 2016 data only, because of the higher statistical precision in the response matrix, which is obtained using simulated $\hbox {t}\overline{\hbox {t}}$ events. Simulated background processes are used to estimate the amount of background events and are subtracted from data. The corresponding statistical uncertainties in the background samples are much smaller than the uncertainties in the cross sections of these processes, which are 19% for production, 23% for single top quark production and 100% for other SM backgrounds [99–104]. The statistical uncertainties from the limited size of the MC samples are found to be a factor of more than three smaller compared to the ones from data.

Model uncertainties arise from the choice of parameters in the event simulation. These parameters include the factorisation and renormalisation scales $\mu _{\textrm{F}}$ and $\mu _{\textrm{R}}$, the top quark mass, the colour reconnection, the UE tune, and the choice of PDFs. Uncertainties in the parton shower are estimated by changing the energy scales for the ISR and the FSR, and varying the parameter that controls the matching between matrix element and parton shower ($h_{\textrm{damp}}$) [69]. These variations cover all observed differences between data and simulation in distributions relevant for this measurement. The uncertainty in the fragmentation of the b quark has been estimated by changing its $p_{\textrm{T}}$ distribution in the powheg +pythia$\hbox {t}\overline{\hbox {t}}$ simulation. It was found to have a negligible effect.

We do not consider an additional uncertainty from a comparison to an alternative parton shower simulation, as for example implemented in the herwig event generator. Simulated $\hbox {t}\overline{\hbox {t}}$ events using powheg +herwig [97] version 7.1 with tune CH3 [105] do not describe the data as well as events produced with powheg +pythia. Furthermore, an uncertainty derived from the difference between these simulations would result in an overestimation of the parton shower uncertainty and in a double counting of uncertainty sources. Instead, accounting for the different sources of parton shower uncertainties (ISR, FSR, $h_{\textrm{damp}}$) provides a means to trace the relevant modelling uncertainties for this measurement. All model parameters are varied within their uncertainties and the corresponding uncertainties in the $m_{\textrm{jet}}$ measurement are estimated as described in the following.

The values of $\mu _{\textrm{F}}$, $\mu _{\textrm{R}}$, and the ISR scales are varied by factors from 0.5 to 2. The parameter $h_{\textrm{damp}}$ and the UE tune are varied within their uncertainties [69]. For $\mu _{\textrm{F}}$ and $\mu _{\textrm{R}}$, there are eight possible combinations to vary the scales. We find that the simultaneous up and down variations of both scales have the largest effects. In order to estimate the uncertainty in the $\mu _{\textrm{F}}$ and $\mu _{\textrm{R}}$ scales, we thus only consider simultaneous shifts of $\mu _{\textrm{F}}$ and $\mu _{\textrm{R}}$. In order to estimate the uncertainty in the colour reconnection model, three different models [106–108] are considered as variations. The uncertainty due to the choice of PDFs has been found to be negligible in the last measurements of $m_{\textrm{jet}}$ [35, 36] because $m_{\textrm{jet}}$ in fully merged top quark decays is sensitive to the decay of the top quark, but not to the dynamics of its production. Therefore, we do not follow the recommendation for estimating PDF uncertainties using different PDF sets [109], but we estimate the PDF uncertainty by using 100 variations of the NNPDF sets versions 3.0 [65] and 3.1 [66].

For all model variations, the simulated $m_{\textrm{jet}}$ distribution at the detector level is unfolded to the particle level using the same setup as for data. Differences between the true distribution at the particle level and the unfolded simulation with model variations indicate a potential bias in the unfolding setup and are treated as uncertainties. For uncertainties in the ISR scale, the $\mu _{\textrm{F}}$ and $\mu _{\textrm{R}}$ scales, the $h_{\textrm{damp}}$ parameter, and the UE tune the average bias of the up and down variations is calculated in each bin and taken as an uncertainty. In the case of the colour reconnection model, the impact of a change in the model is calculated by taking the difference in the mean of $m_{\textrm{jet}}$ between the true distribution at the particle level and the unfolded distribution. The model with the largest difference is chosen, and we take the resulting bias as the uncertainty from the colour reconnection model.

**Fig. 9:**
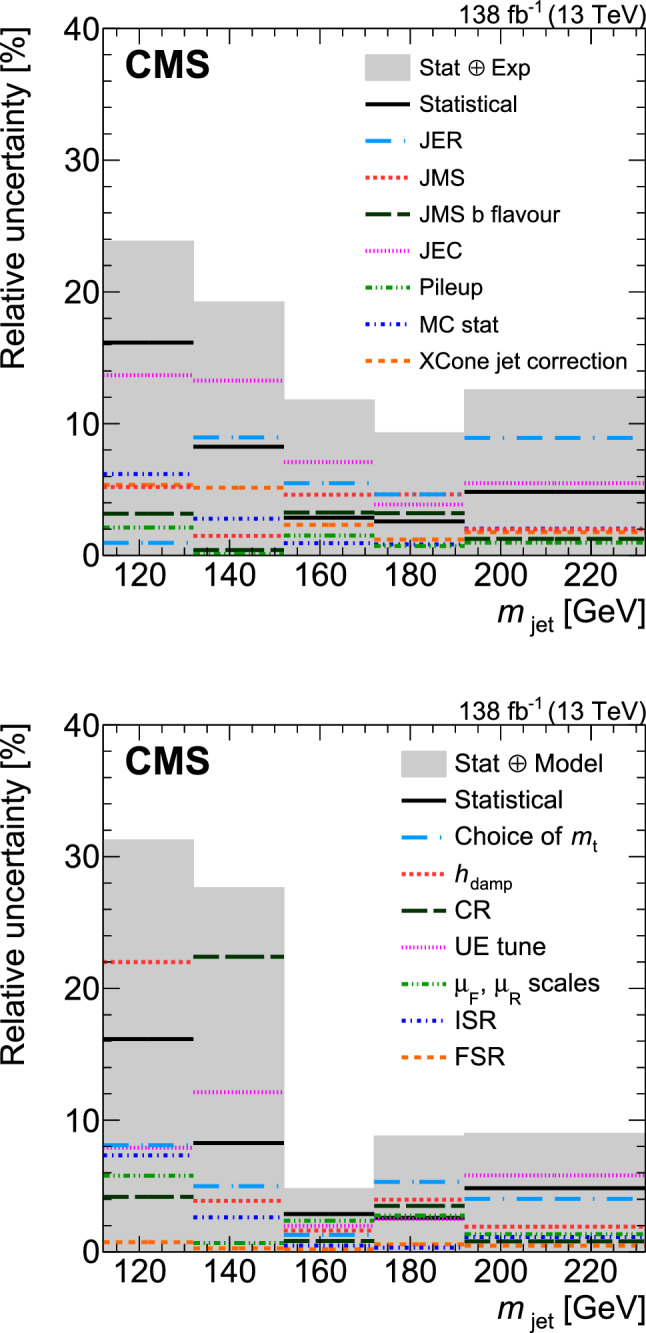
Relative experimental (upper) and model (lower) uncertainties in the measurement of $m_{\textrm{jet}}$. Various sources are displayed as coloured lines and compared to the total experimental or model uncertainty, respectively. The uncertainty sources are calculated as the square root of the diagonal entries from the respective covariance matrix, and do not include bin-to-bin correlations

The uncertainty due to the choice of $m_{\textrm{t}}$ in the $\hbox {t}\overline{\hbox {t}}$ simulation used to unfold the data is calculated using samples with different values of $m_{\textrm{t}}$. The difference between the unfolded distribution and the true particle-level distribution is parametrised in each bin of the unfolded distribution. We use a linear function with $m_{\textrm{t}}$ as its argument to describe the difference. The parameters of this function are obtained using the $\hbox {t}\overline{\hbox {t}}$ samples with $m_{\textrm{t}} = 169.5$, 171.5, 173.5, and 175.5$\,\text {Ge}\hspace{-.08em}\text {V}$. The uncertainty is then evaluated from the linear function at $m_{\textrm{t}} = 172.5 \pm 1\,\text {Ge}\hspace{-.08em}\text {V}$. This procedure has the advantage of being less susceptible to statistical fluctuations in the individual samples, therefore resulting in a more reliable estimate of this uncertainty. The interval of ${\pm }1\,\text {Ge}\hspace{-.08em}\text {V}$ has been found to be sufficient, because larger variations do not agree with the data at the detector level.

We use the same method to calculate the uncertainty in the modelling of FSR. The simulated samples with different choices of $f_{\textrm{FSR}}$ are unfolded, and the differences between the true distribution at the particle level and the unfolded distributions are parametrised as a function of $f_{\textrm{FSR}}$ in each bin. The uncertainty is obtained by evaluating the parametrisation at the values obtained in the studies described in Sect. 8.

Figure 9 summarises the experimental and model uncertainties in the measurement of $m_{\textrm{jet}}$. The largest experimental uncertainties arise from the JES and JER corrections. In the $m_{\textrm{jet}}$ peak region (the third and fourth bins) the largest sources of model uncertainties are from the UE tune, the $h_{\textrm{damp}}$ parameter, and the choice of $m_{\textrm{t}}$. In the first two bins, the limited statistical precision of the samples with model variations, in combination with a smaller number of observed events than in the peak region, leads to statistical fluctuations in the estimation of model uncertainties. This results in large uncertainties from $h_{\textrm{damp}}$ and the colour reconnection models in the first and second bins of the measurement, respectively. Because the sensitivity to $m_{\textrm{t}}$ of the $m_{\textrm{jet}}$ measurement comes from the peak region, these uncertainties have a minor effect on the determination of $m_{\textrm{t}}$.

**Fig. 10:**
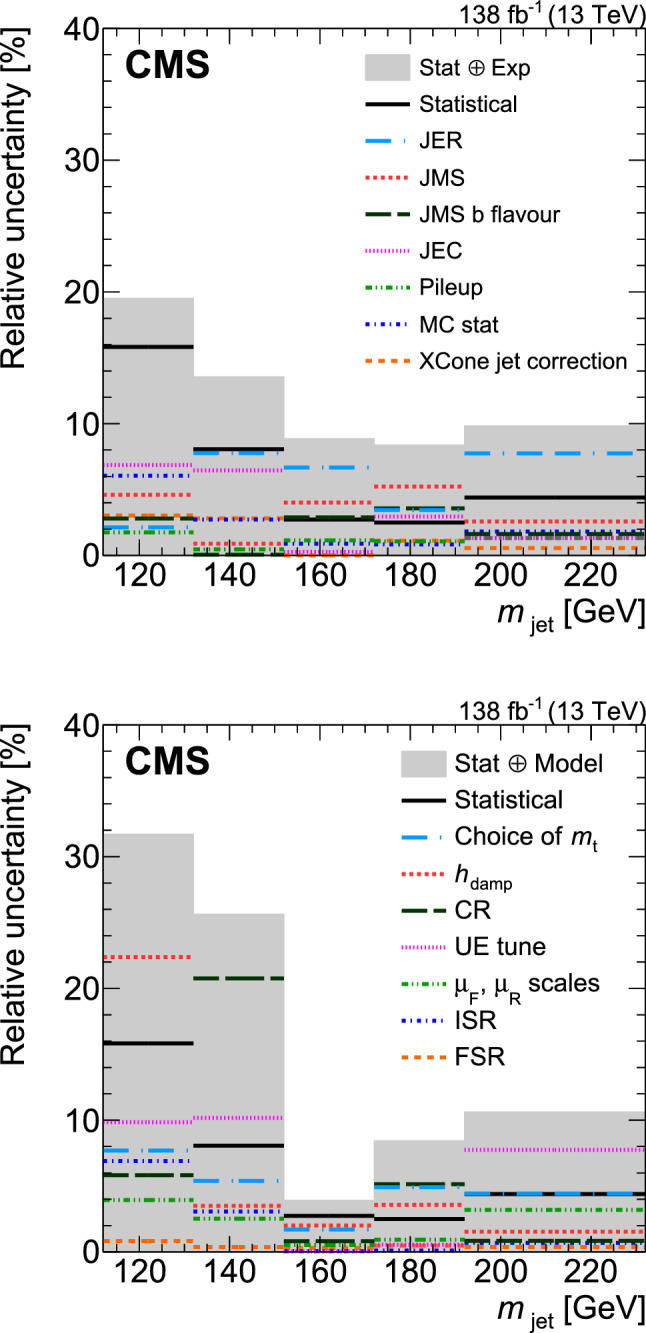
Relative experimental (upper) and model (lower) uncertainties after normalising the measurement to the total cross section. Various sources are displayed as coloured lines and compared to the total experimental or model uncertainty, respectively. The uncertainty sources are calculated as the square root of the diagonal entries from the respective covariance matrix, and do not include bin-to-bin correlations

When normalising the unfolded distribution, systematic uncertainties cancel fully or partially. For example, the uncertainty in the integrated luminosity cancels completely as it affects all bins by an equal amount. The uncertainty component in the JEC that changes only the three-vector predominantly changes the XCone jet $p_{\textrm{T}}$, and thus affects the selection efficiency of the measurement. This uncertainty cancels to a large part when normalising the measurement and becomes negligible. The uncertainties in the normalised measurement are summarized in Fig. 10. In the peak region, the dominant experimental uncertainties originate from JER and JMS corrections. The dominant model uncertainties are the same as for the absolute cross section measurement.

Theory uncertainties are those uncertainties that apply to predictions at the particle level. The scales for FSR, ISR, as well as $\mu _{\textrm{F}}$ and $\mu _{\textrm{R}}$, are varied by factors of 0.5 and 2. The UE tune and the $h_{\textrm{damp}}$ parameter are varied within their uncertainties. All three models of colour reconnection are used to calculate the corresponding uncertainty. For each source, the uncertainty in each bin is estimated by the largest difference to the nominal prediction at the particle level.

## Results and determination of the top quark mass

**Fig. 11:**
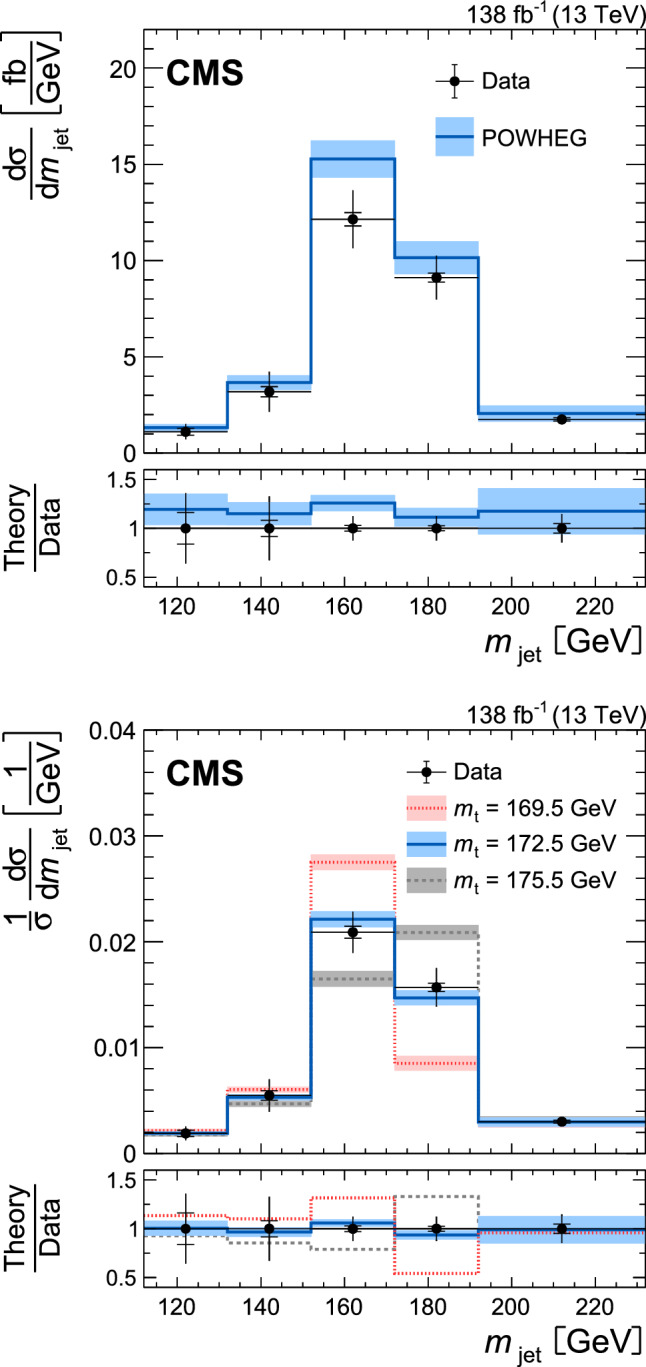
Differential $\hbox {t}\overline{\hbox {t}}$ production cross section as a function of $m_{\textrm{jet}}$ compared to predictions obtained with powheg: absolute (upper) and normalised (lower). For the normalised measurement, the data are compared to predictions with different $m_{\textrm{t}}$. The vertical bars represent the total uncertainties, and the statistical uncertainties are shown by short horizontal bars. The long horizontal bars reflect the bin widths. Theoretical uncertainties in the prediction are indicated by the bands. The lower panels show the ratio of the theoretical prediction to data

The three different years, as well as the electron and muon channels, are combined before the unfolding, but are also processed individually to validate their consistency. Figure 11 (upper) shows the differential $\hbox {t}\overline{\hbox {t}}$ cross section in the fiducial region as a function of $m_{\textrm{jet}}$, measured in data and compared to simulation. The $\hbox {t}\overline{\hbox {t}}$ production cross section in the fiducial region is measured to be $581 \pm 8 \,\text {(stat)} \pm 46 \,\text {(exp)} \pm 19 \,\text {(model)} \,\text {fb}$. This can be compared to the prediction from the powheg simulation, $690 \pm 59\,\text {fb}$. The smaller value of the measured cross section compared to the prediction from powheg at NLO has been observed in other analyses for top quark $p_{\textrm{T}} >400\,\text {Ge}\hspace{-.08em}\text {V}$ [90–92], where NNLO calculations describe the shape of the top quark $p_{\textrm{T}}$ distribution better.

We determine the value of $m_{\textrm{t}}$ from the normalised differential $\hbox {t}\overline{\hbox {t}}$ production cross section as a function of $m_{\textrm{jet}}$. This enables a measurement using the shape of the $m_{\textrm{jet}}$ distribution without sensitivity to uncertainties in the normalisation. Figure 11 (lower) shows the normalised measurement compared to predictions from powheg with different values of $m_{\textrm{t}}$. In order to extract $m_{\textrm{t}}$, a fit is performed based on $\chi _m^2 = d_{m} ^T V_{m} ^{-1} d_{m}$, where $d_{m}$ is the vector of differences between the measured normalised differential cross section and the powheg simulation with different values of $m_{\textrm{t}}$. Four of the five bins in $m_{\textrm{jet}}$ are used in the calculation of $d_{m}$, because of the normalisation of the measurement. The covariance matrix $V_{m}$ contains all statistical, experimental, model, and theory uncertainties. We use the Linear Template Fit [110] package to parametrise the cross section as a function of $m_{\textrm{t}}$ and obtain the best fit value with the corresponding uncertainties analytically.

**Fig. 12:**
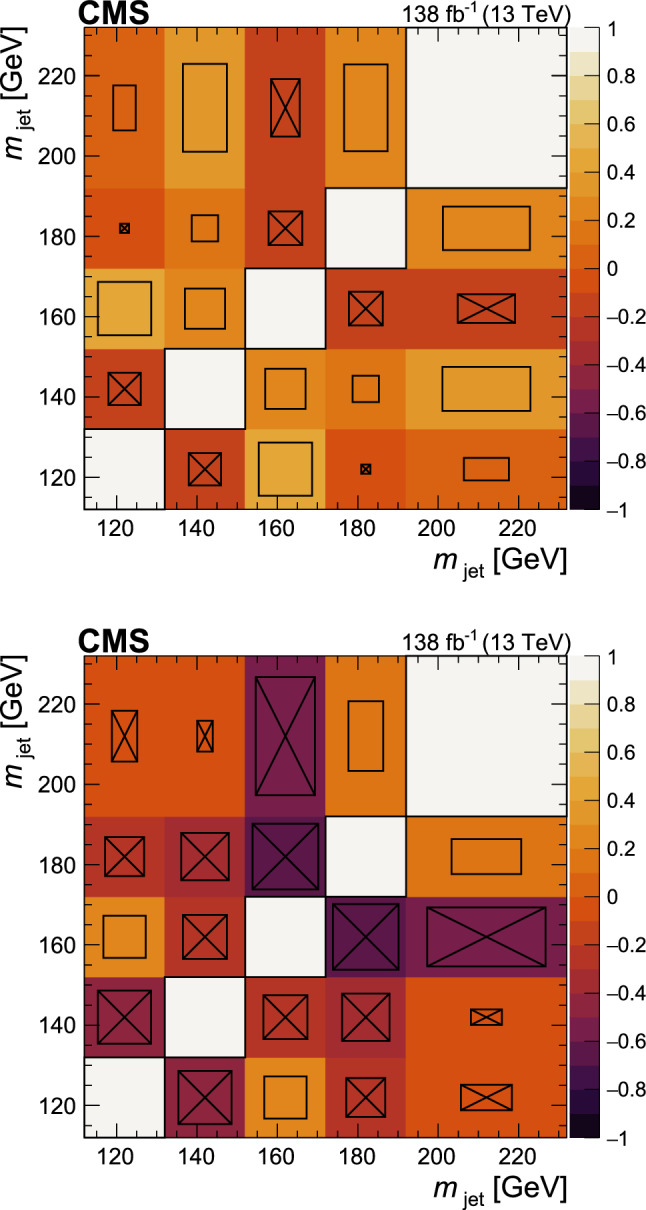
Correlations between the bins in the unfolding before (upper) and after (lower) normalising the distribution to the total cross section. Boxes with crosses indicate negative values of the correlation coefficient

The bin-to-bin correlations in the measurement calculated from $V_{m}$, including statistical, experimental, and model contributions, are displayed in Fig. 12. Negative correlations between neighbouring bins originate from migrations at the detector level, which have been corrected for by the unfolding and result in anticorrelated statistical uncertainties. The systematic variations that shift the peak of the $m_{\textrm{jet}}$ distribution, for example the JMS, also contribute to the negative correlations.

**Fig. 13:**
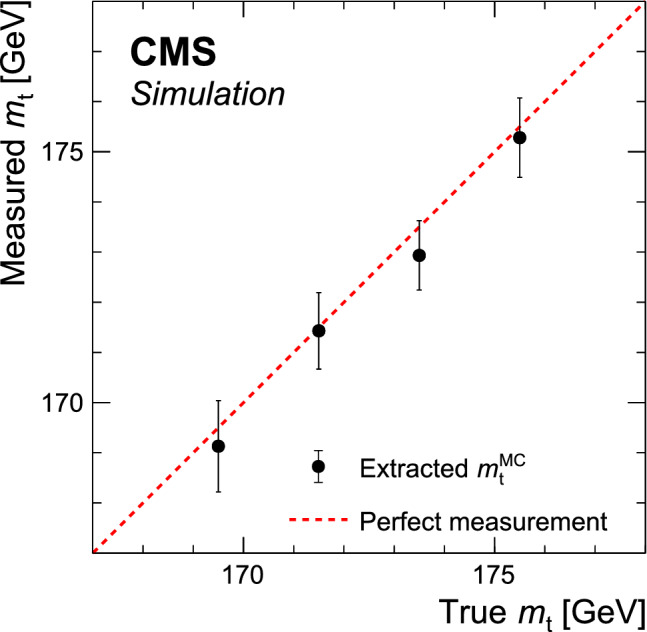
Extracted top quark mass from simulation compared to the true value. The vertical error bars show the total uncertainty in the extraction of $m_{\textrm{t}}$

In order to validate that the determination of $m_{\textrm{t}}$ is unbiased, we perform the $m_{\textrm{t}}$ measurement using simulated samples with various values of $m_{\textrm{t}}$. The obtained value of $m_{\textrm{t}}$ is compared to the true value in Fig. 13. In this comparison, all extracted values agree with the respective true values of $m_{\textrm{t}}$, demonstrating the validity of the mass extraction.

Performing the extraction on collision data and considering all sources of uncertainties, we extract $m_{\textrm{t}}$ using the powheg +pythia simulation,

$$\begin{aligned} \begin{aligned} m_{\textrm{t}}&= 173.06 \pm 0.24\,\text {(stat)} \pm 0.61 \,\text {(exp)} \\&\quad \pm 0.47 \,\text {(model)} \pm 0.23 \,\text {(theo)} \,\text {Ge}\hspace{-.08em}\text {V} \\&= 173.06 \pm 0.84 \,\text {Ge}\hspace{-.08em}\text {V}. \end{aligned} \end{aligned}$$

With respect to the previous CMS measurement at 13$\,\text {Te}\hspace{-.08em}\text {V}$ [36], this corresponds to an improvement by more than a factor of three in terms of precision. This measurement from boosted top quark production has an uncertainty comparable with the most precise $m_{\textrm{t}}$ extractions from fully resolved final states [9–15].

When unfolding the 2016, 2017 and 2018 data separately and extracting $m_{\textrm{t}}$ from these three independent measurements, we find agreement between the extracted values of $m_{\textrm{t}}$ to better than one standard deviation. All three values are compatible with the combined value to better than one half standard deviation. We find the same when unfolding the electron and muon channels separately.

**Table 1:** Total and individual uncertainties in the extraction of $m_{\textrm{t}}$ from the normalised differential cross section. The uncertainties are grouped into experimental, model, theory, and statistical uncertainties. Uncertainties from the choice of the PDF, b tagging, the luminosity measurement, and the lepton triggers, identification and reconstruction are smaller than 0.01$\,\text {Ge}\hspace{-.08em}\text {V}$ and are not listed

Source	Uncertainty [$\text {Ge}\hspace{-.08em}\text {V}$]
Jet energy resolution	0.38
Jet mass scale	0.37
Jet mass scale b flavour	0.26
MC stat	0.09
Pileup	0.08
Jet energy scale	0.07
Additional XCone corrections	0.01
Backgrounds	0.01
Experimental total	0.61
Choice of $m_{\textrm{t}}$	0.41
Colour reconnection	0.17
$h_{\textrm{damp}}$	0.09
Underlying event tune	0.09
$\mu _{\textrm{F}}$, $\mu _{\textrm{R}}$ scales	0.08
ISR	0.02
FSR	0.02
Model total	0.47
Underlying event tune	0.13
FSR	0.11
$\mu _{\textrm{F}}$, $\mu _{\textrm{R}}$ scales	0.10
Colour reconnection	0.09
$h_{\textrm{damp}}$	0.04
ISR	0.04
Theory total	0.23
Statistical	0.24
Total	0.84

The individual sources of uncertainty and their impact on the mass extraction are detailed in Table 1. The dominant experimental uncertainties are connected to the calibration of the JER, the JMS calibration, and the JMS b flavour uncertainty, also visible in Fig. 10. The dominant modelling uncertainties arise from the choice of the $m_{\textrm{t}}$ and $h_{\textrm{damp}}$ parameters in the $\hbox {t}\overline{\hbox {t}}$ simulation. Compared to the previous measurement, the dedicated measurement of the JMS leads to an uncertainty reduced by a factor of 5 in the jet calibration. By constraining the simulation of FSR with data, this previously dominant model uncertainty becomes small. The use of about four times the data, corresponding to an integrated luminosity of 138$\,\text {fb}^{-1}$, leads to a reduction in the statistical uncertainty by a factor of 2.

The improvements described in this article result in a considerable gain in precision, allowing for a determination of $m_{\textrm{t}}$ from $\hbox {t}\overline{\hbox {t}}$ production at high $p_{\textrm{T}}$ with an uncertainty comparable to the one achieved in measurements close to the $\hbox {t}\overline{\hbox {t}}$ production threshold with fully resolved final state objects. The measurement also provides important information on the modelling of the jet mass in decays of boosted top quarks, which is the most important substructure variable for the identification of large-radius jets [111].

## Conclusions

A measurement of the differential top quark pair ($\hbox {t}\overline{\hbox {t}}$) production cross section as a function of the jet mass $m_{\textrm{jet}}$ in hadronic decays of boosted top quarks has been presented. The normalised distribution in $m_{\textrm{jet}}$ is sensitive to the top quark mass $m_{\textrm{t}}$, which is measured to be $173.06 \pm 0.84\,\text {Ge}\hspace{-.08em}\text {V}$. This value is compatible with earlier precision measurements in fully resolved final states [11, 14, 15]. With respect to an earlier CMS analysis [36], the precision is improved by a factor of more than three. This has been achieved by a dedicated calibration of the jet mass scale, a study of the effects of final state radiation inside large-radius jets, and about 4 times more data. With these improvements, the uncertainty in the extraction of $m_{\textrm{t}}$ at high top quark boosts becomes comparable to direct measurements close to the $\hbox {t}\overline{\hbox {t}}$ production threshold. The sources of the leading systematic uncertainties are very different, highlighting the complementarity of this measurement. In addition, the study of boosted top quarks offers the possibility to directly compare the distribution in $m_{\textrm{jet}}$ to analytic calculations [34]. When these calculations become available, the unfolded $m_{\textrm{jet}}$ distribution can be used to measure the top quark pole mass directly. The precisely measured differential cross section as a function of $m_{\textrm{jet}}$ represents an important step towards understanding and resolving the ambiguities between the top quark mass extracted from a direct reconstruction of $m_{\textrm{t}}$, and the top quark pole mass.

## Data Availability

This manuscript has no associated data or the data will not be deposited. [Authors’ comment: Release and preservation of data used by the CMS Collaboration as the basis for publications is guided by the CMS policy as stated in https://cms-docdb.cern.ch/cgibin/PublicDocDB/RetrieveFile?docid=6032 &filename=CMSDataPolicyV1.2.pdf &version=2.]
